# Tropical Montane Cloud Forests Have High Resilience to Five Years of Severe Soil Drought

**DOI:** 10.1111/gcb.70670

**Published:** 2026-01-07

**Authors:** David C. Bartholomew, Paulo R. L. Bittencourt, Darcy Galiano Cabrera, Roxana Sacatuma Cruz, Jimmy R. Chambi Paucar, Daniela Corrales Alvarez, Eric Cosio, Blanca Espinoza Otazu, Darwin Manuel Mamani, Patrick Meir, George A. Muñoz Hermoza, Rafael S. Oliveira, Beisit L. Puma Vilca, Aida Rosalai, Carlos Salas Yupayccana, Norma Salinas, José Sanchez Tintaya, Jhon A. Yuca Palomino, Daniel B. Metcalfe

**Affiliations:** ^1^ Department of Ecology and Environmental Science Umeå University Umeå Sweden; ^2^ Botanic Gardens Conservation International Richmond UK; ^3^ School of Earth and Environmental Sciences Cardiff University Cardiff UK; ^4^ ABIDA Cuzco Peru; ^5^ Universidad Nacional de San Antonio Abad del Cusco Cusco Peru; ^6^ Pontifical Catholic University of Peru San Miguel Peru; ^7^ University of Edinburgh Edinburgh UK; ^8^ Departamento de Biologia Vegetal Universidade Estadual de Campinas (UNICAMP) Campinas Brazil

**Keywords:** carbon cycling, climate change, drought, hydraulics, leaf nutrients, non‐structural carbohydrates, photosynthesis, respiration, throughfall exclusion, tropical montane cloud forests

## Abstract

Tropical montane cloud forests (TMCFs) are globally important ecosystems, acting as large carbon sinks and supporting exceptional biodiversity. However, climate‐driven declines in rainfall threaten these forests, but their responses to long‐term soil moisture deficit remain poorly understood. We implemented a 5‐year throughfall exclusion (TFE) experiment in a Peruvian TMCF, reducing soil moisture by 69.1% across a 0.09 ha plot. We compared the full carbon cycle budget, and surveyed tree physiological traits linked to hydraulics, metabolism and nutrients in the TFE plot and an adjacent, unmodified control (CON) plot. Soil drought reduced gross primary productivity by 4.24 ± 1.97 Mg C ha^−1^ year^−1^ but did not change net primary productivity because of an equivalent 3.38 ± 1.42 Mg C ha^−1^ year^−1^ decline in autotrophic respiration. Net ecosystem exchange also remained unchanged over 5 years of soil drought. Trees did not change xylem conductivity, hydraulic safety margins or photosynthetic capacity in the TFE, but did have 0.027 ± 0.011 g cm^−3^ denser wood and 4.58% ± 1.03% higher trunk starch concentrations. These results suggest that trees in TMCF avoid hydraulic failure and carbon starvation under sustained soil moisture drought via metabolic downregulation, resource conservation and non‐structural carbohydrate storage. However, reduced uptake of nutrients (nitrogen, phosphorus, calcium) and 90.6% ± 29.8% decline in fruit production may impact future growth and demography. Our findings demonstrate surprising resilience of TMCFs to sustained, severe soil drought but highlight potential impacts on nutrient cycling and reproduction under climate change. Understanding the impacts of soil drought in conjunction with other climatic changes (e.g., fog reduction, temperature increases) is needed to fully assess the resilience of TMCFs to climate change.

## Graphical abstract

A 5‐year throughfall exclusion experiment in a Peruvian tropical montane cloud forest reduced soil moisture by 69%, leading to lower photosynthetic inputs and autotrophic respiration but maintaining net primary productivity and carbon sink capacity. Trees responded through metabolic downregulation, reduced fruit production, increased wood density, and starch accumulation while maintaining hydraulic stability.

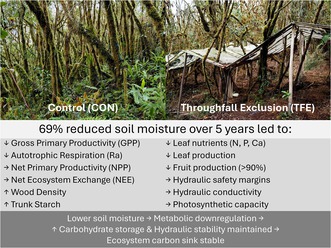

## 1 Introduction

Tropical montane cloud forests (TMCF) are rare ecosystems, making up only 2.5% of the area of tropical forests worldwide (Bubb et al. 2004). Nevertheless, these forest types are extremely valuable as biodiversity hotspots (Bubb et al. 2004; Olson and Dinerstein 2002), carbon stores (Spracklen and Righelato 2014) and as regulators of freshwater supplies (Bruijnzeel et al. 2011; Bruijnzeel and Hamilton 2000). These ecosystem functions are intimately tied to the high frequency of cloud immersion, which typifies TMCF (Bruijnzeel et al. 2011; Goldsmith et al. 2013), and the abundance and diversity of plant species in TMCF clearly adapted to this immersion (Benzing 1998). However, the extent and frequency of cloud immersion across TMCF‐dominated regions are projected to decline drastically over the next half century because of climate change and regional land use changes (Guzmán Q et al. 2024; Helmer et al. 2019; Still et al. 1999). Warming is expected to increase the mean elevation at which clouds form, and regional deforestation around mountains is expected to reduce the supply of moisture to air, which subsequently would produce clouds travelling upwards along the mountain slope (Guzmán Q et al. 2024; Helmer et al. 2019; Still et al. 1999). Since TMCFs rarely experience severe soil water deficit, they may be poorly adapted to survive these changes, but our knowledge of the climatic sensitivity of TMCFs still lags far behind other ecosystems.

As the frequency and severity of drought have increased across many tropical regions (Chadwick et al. 2016; Malhi et al. 2009; Zhang et al. 2015), the incentive to develop a clearer predictive understanding of likely ecosystem responses to these climatic shifts has grown. One important advance has been the establishment of large‐scale rainfall manipulation experiments in lowland tropical forests (Asbjornsen et al. 2018; Bonal et al. 2016; Knapp et al. 2024). For example, at a site in the eastern Amazon, trees responded to drought by allocating more C below‐ground, and to respiration rather than biomass growth (Da Costa et al. 2010; Metcalfe et al. 2010). Photosynthesis was suppressed more than total respiration, such that the net C sink declined following drought (Doughty et al. 2015; Metcalfe et al. 2010). Nevertheless, tree mortality only increased after ~7 years of severe (50% reduction), sustained drought, and then because of increased hydrological stress not due to any scarcity in carbohydrate reserves (Bittencourt et al. 2020; Rowland, da Costa, et al. 2015). Wider replication of similar experiments around the tropical lowlands (Asbjornsen et al. 2018) has provided critical general insights into how drought impacts lowland forest carbon cycling, greatly improving the representation of drought impacts in ecosystem models (McDowell et al. 2013; Powell et al. 2013; Zhou et al. 2019).

Compared to the lowland tropics, contemporary scientific understanding of TMCFs has benefited little from experimental manipulations of climatic factors of interest. To our knowledge, there have been no large‐scale manipulations of incident rainfall in TMCF. Although researchers have investigated climatic sensitivity of TMCF species via other approaches, such as observations over time (Foster 2001; Pounds et al. 1999), measurements along natural environmental gradients (Girardin et al. 2010), and transplantation of plants to sites with different levels of fog immersion (Nadkarni and Solano 2002; Rapp and Silman 2014). Further, the different forms of water inputs (rainfall and fog) predominant in TMCF, together with the distinct ecology and biogeochemistry of the system (Fahey et al. 2016; Gotsch et al. 2016; Willig and Presley 2016), preclude direct inferences of experimental results from lowland forest to TMCF. For example, the presence of fog and humid conditions coupled with low temperatures and short statured trees may mean that TMCF trees are more resilient to soil drought than trees in lowland forests (Eller et al. 2013). This may depend in part on the evolutionary origin of the TMCF species—if they are high altitude specialists or originate from lowland tropical or temperate taxa (Barros et al. 2022). Alternatively, the relatively thin soils and limited exposure to previous droughts may mean that TMCF are particularly vulnerable (Foster 2001). Indeed, studies transplanting plants adapted to cloud forest conditions downslope to less cloudy conditions have generally shown declines in plant survival and growth (Nadkarni and Solano 2002; Rapp and Silman 2014). Further, how these responses to water stress are manifested—via changes in plant hydraulics and/or carbon economy (McDowell 2011)—and the wider implications for ecosystem C storage and net uptake remain unknown.

Therefore, the purpose of this study was to present an overview of ecosystem impacts from the first large‐scale throughfall exclusion (TFE) experiment imposed in TMCF. In the present analysis, we present data from the first 62 months following the treatment installation, compared throughout with a nearby, unmodified control plot (CON). While the TFE treatment was not replicated (Hurlbert 1984, 2004), it provides insights into ecosystem processes that would otherwise have been impossible to capture in smaller‐scale experiments (Carpenter 1996; Osmond et al. 2004; Stokstad 2005; Sullivan 1997). On both plots, we collected (i) all major ecosystem carbon stocks and fluxes to estimate ecosystem‐level carbon cycling, allocation and net uptake, and (ii) functional traits linked to ecophysiology, hydraulics, carbon and nutrient economy to provide information about plant drought adaptation strategies. Finally, we compare these results to matching information from a nearby cloud water reduction experiment (Metcalfe et al. 2025; Bartholomew, D. C., Bittencourt, P. R. L., Galiano Cabrera, D., Sacatuma Cruz, R., Asbjornsen, H., Brum, M., Chambi Paucar, J. R., Corrales Alvarez, D., Cosio, E., Espinoza Otazu, B., Mamani, D. M., Meir, P., Muñoz Hermoza, G. A., Oliveira, R. S., Puma Vilca, B. L., Rosalai, A., Salas Yupayccana, C., Salinas, N., Sanchez Tintaya, J., Vadeboncoeur, M. A., Yuca Palomino, J. A., Metcalfe, D. B., unpublished data) to provide insights into the effects of shifts in the forms of water inputs to the ecosystem.

## 2 Materials and Methods

### 2.1 Study Site

**FIGURE 1:**
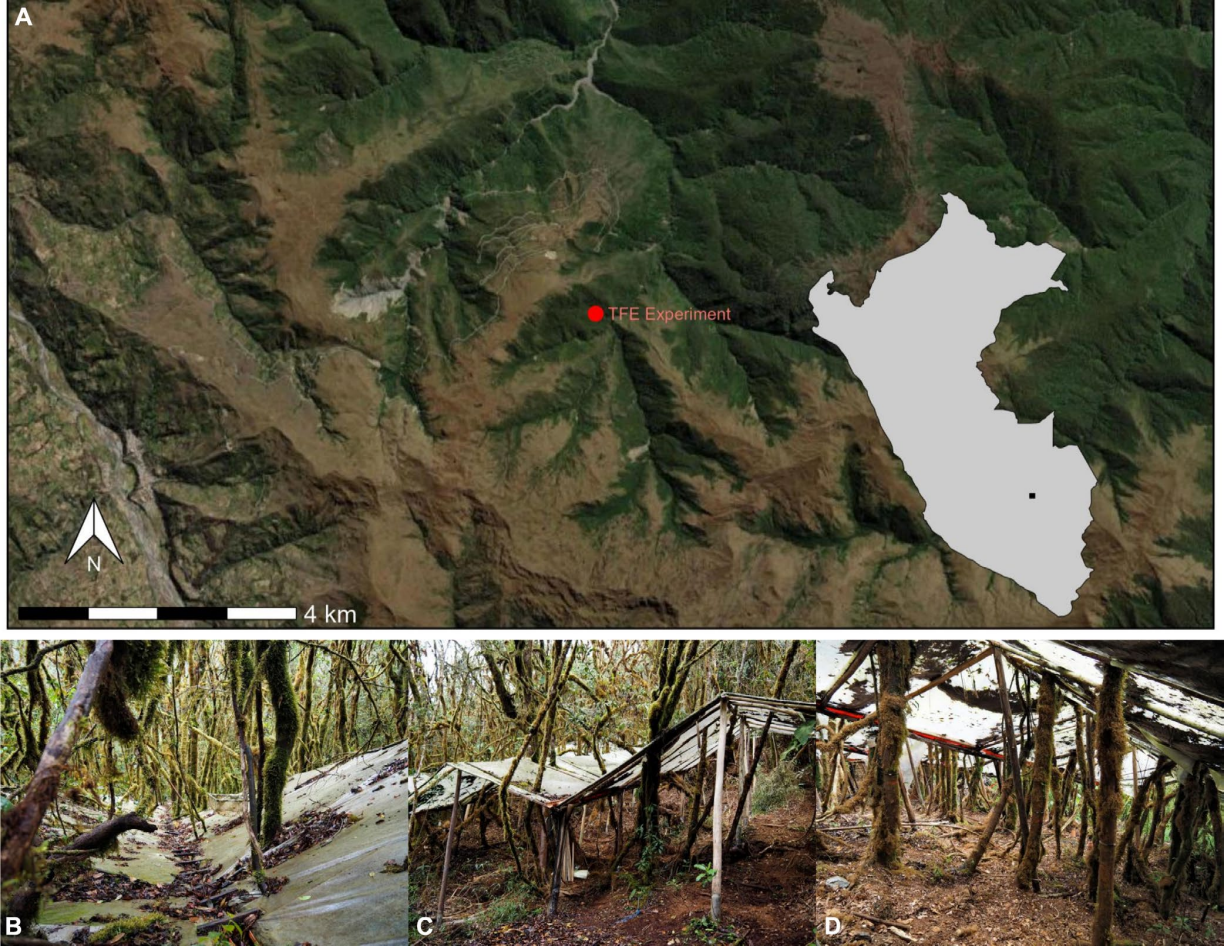
Location and design of the throughfall exclusion (TFE) experiment. (A) Map showing the location of the study site in the Kosñipata Valley. (B–D) Photos showing the infrastructure of the throughfall exclusion (TFE) experiment from above the plastic panels (B), outside the experiment (C) and below the plastic panels (D). Photo credit: David C. Bartholomew. Map lines delineate study areas and do not necessarily depict accepted national boundaries.

This study was conducted in a Tropical Montane Cloud Forest (TMCF) within the Kosñipata catchment, situated on the windward slope of the Peruvian Andes. The region experiences persistent cloud immersion, driven by the collision of cold Andean air masses with warm, moisture‐laden air from the Amazon lowlands (Killeen et al. 2007). High humidity and frequent rainfall define the climate, with an average annual precipitation of approximately 1500 mm. Water inputs to the catchment are primarily from direct rainfall (90.8%), with cloud‐derived moisture contributing 9.2%, though these proportions vary seasonally and across elevations (Clark et al. 2014). Local recycling accounts for around 60% of atmospheric moisture input (Horwath et al. 2019), highlighting the importance of regional hydrological dynamics. The forest is structurally complex, with a closed canopy and a sparse understory, while tree trunks and branches are densely colonised by epiphytes, including bryophytes (mosses and liverworts), ferns, orchids, bromeliads and Ericaceae species (Horwath et al. 2019). The throughfall exclusion (TFE) experiment is situated approximately 1 km from the Wayqecha Biological Station in the Kosñipata catchment (−13.17495, −71.58716) at an elevation of ~3000 m above sea level and is adjacent to the Wayqecha (WAY‐01) permanent plot, part of the Andes Biodiversity and Ecosystems Research Group (ABERG) network (Malhi et al. 2010). The experiment comprises two adjacent plots: a TFE plot (0.09 ha), where 98% of the ground surface is covered with clear plastic panels installed at ~2 m height since 2017, and a control plot (0.09 ha) located 20 m away (Figure 1). Site selection ensured both plots (i) exhibited no evidence of human disturbance, such as logging or fire, and (ii) had comparable species composition and biomass prior to the treatment initiation. To minimise potential nutrient cycle disruptions, accumulated litterfall was removed from the plastic panels every month and placed on the forest floor. The change in soil moisture between the CON and TFE was measured with 11 sensors (5 in CON; 6 in TFE; TMS‐4 datalogger, Tomst s.r.o., Michelská, Czech Republic). On average, the TFE reduced soil moisture by 69.12% (Figure S1).

### 2.2 Carbon Fluxes

All key ecosystem carbon fluxes were measured over a 62‐month period, from November 2017 to December 2022, using the Global Ecosystem Monitoring protocol (Malhi et al. 2021), a standardised approach extensively applied in tropical forests, including TMCFs (Girardin, Farfan‐Rios, et al. 2014). These measurements were applied both to the TFE and CON plots, and a nearby cloud reduction experiment (Metcalfe et al. 2025; Bartholomew, D. C., Bittencourt, P. R. L., Galiano Cabrera, D., Sacatuma Cruz, R., Asbjornsen, H., Brum, M., Chambi Paucar, J. R., Corrales Alvarez, D., Cosio, E., Espinoza Otazu, B., Mamani, D. M., Meir, P., Muñoz Hermoza, G. A., Oliveira, R. S., Puma Vilca, B. L., Rosalai, A., Salas Yupayccana, C., Salinas, N., Sanchez Tintaya, J., Vadeboncoeur, M. A., Yuca Palomino, J. A., Metcalfe, D. B., unpublished data). Gross primary productivity (GPP) was estimated by summing net primary productivity (NPP) and autotrophic respiration (*R*_a_), while carbon use efficiency (CUE) was calculated as the proportion of GPP allocated to NPP. Net Ecosystem Exchange (NEE) was derived by subtracting heterotrophic respiration (*R*_h_) from NPP. Above‐ground NPP was estimated as the sum of multiple components: coarse wood production (NPP_ACW_), branch turnover (NPP_branch turnover_), litterfall (NPP_litterfall_) and biomass loss due to leaf herbivory (NPP_herbivory_). To assess autotrophic (*R*_a_) and heterotrophic (*R*_h_) respiration, we quantified key components of ecosystem respiration in both plots, including canopy respiration (*R*_leaves_), above‐ground live wood respiration (*R*_stems_), coarse root respiration (*R*_coarse roots_) and total soil CO_2_ efflux (*R*_soil_), which was further partitioned into its autotrophic (*R*_rhizospere_) and heterotrophic (*R*_het_) components. See Supporting Information for more details of how specific components were calculated. Due to the absence of replication in the experimental design, formal statistical comparisons were not possible (Hurlbert 1984, 2004), but 95% confidence intervals were used to assess the reliability of observed differences at the plot level. Error bars presented in the manuscript represent within‐plot spatial variability and measurement uncertainty rather than landscape‐scale variation. Error propagation followed standard approaches, applying the quadrature of absolute errors for sums and differences, and the quadrature of relative errors for ratios and products, under the assumption that errors were independent and normally distributed (Aragão et al. 2009).

### 2.3 Trait Sampling

A total of 79 trees were sampled across the two plots, with 40 from the CON and 39 from the TFE plot. Sampling focused on the nine most common genera—*Clethra*, *Clusia*, *Meliosma*, *Miconia*, *Myrsine*, *Ocotea*, *Persea*, *Prunus*, *Weinmannia*—which were distributed across both plots, except for *Miconia*, which was only present in the CON plot. Tree selection aimed to include up to five individuals per species, with replication occurring at the genus level when species‐level replication was not possible. Sampling was conducted during the peak wet season (January–March 2022) to assess gas exchange, leaf morphology, leaf nutrient content and non‐structural carbohydrate traits. A second sampling was conducted at the end of the dry season (August–September 2022) to measure hydraulic and wood traits.

### 2.4 Hydraulic Traits and Wood Properties

To assess plant water status and hydraulic function, we measured midday leaf water potential (Ψ_md_), wood capacitance, leaf hydrophobicity, leaf water retention, xylem hydraulic conductance and embolism resistance across both plots. We measured hydraulic vulnerability traits because embolism formation is closely tied to drought mortality in lowland tropical forests (Barros et al. 2019; Bittencourt et al. 2020), and thresholds such as hydraulic safety margins and percentage loss of conductivity indicate whether trees are approaching conditions where mortality risk increases. Midday leaf water potential was recorded using a Scholander pressure chamber. Leaf hydrophobicity and water retention were assessed by analysing droplet contact angles and leaf inclination thresholds (Jurak et al. 2020; Lenz et al. 2022). Hydraulic conductance and native embolism were quantified by measuring xylem conductivity before and after emboli removal, using a hydraulic apparatus to monitor water flow and pressure changes (Bittencourt et al. 2022). Xylem embolism resistance was estimated via the pneumatic method (Pereira et al. 2016, 2020), which tracked air discharge under decreasing water potential. Hydraulic safety margins (HSM_Ψ50_ and HSM_Ψ88_) were calculated to evaluate drought vulnerability. Wood density was determined from rehydrated branch segments using the water displacement method (Pérez‐Harguindeguy et al. 2013). Detailed methodologies are provided in the Supporting Information.

### 2.5 Gas Exchange Measurements

Gas exchange parameters were assessed by measuring photosynthetic capacity and dark respiration (*R*_dark_) on branches collected from the sun‐exposed canopy during the peak wet season (January–March 2021). To maintain hydraulic continuity, branches were cut in the field, re‐cut underwater (Domingues et al. 2010) and transported to the laboratory, where they were stabilised before measurement. Photosynthetic capacity was determined by conducting CO_2_‐response (*A–C*_*i*_) and light‐response (*A–Q*) curves using a portable photosynthesis system (LI‐6400XT; LI‐COR, Nebraska, USA) under controlled environmental conditions. The maximum rate of photosynthesis (*A*_max_), assimilation under saturating light and ambient CO_2_ (*A*_sat_), carboxylation capacity (*V*_cmax_) and electron transport rate (*J*_max_) were estimated from these response curves (Sharkey et al. 2007).

Leaf dark respiration (*R*_dark_) was measured on adjacent leaves after a 30‐min dark adaptation period. Measurements were conducted using a portable photosynthesis system, and respiration values were standardised to 25°C using a *Q*_10_ correction factor (Rowland, Lobo‐do‐Vale, et al. 2015). Stomatal conductance during *R*_dark_ was also recorded to estimate minimum stomatal conductance (*g*_dark_). Full methodological details are provided in the Supporting Information.

### 2.6 Non‐Structural Carbohydrate (NSC) Analysis

Total NSCs were measured in leaves, branches and trunks. Leaf and branch samples were collected during the peak wet season (January–March 2022) from branches adjacent to those harvested for gas exchange measurements. Trunk samples were obtained by extracting the outer 3 cm of wood at 1.3 m height using an increment borer (Haglöf Sweden AB, Långsele, Sweden). Sampling was conducted in the early morning (08:00–10:00) to minimise diurnal variation in NSC concentrations.

Immediately after sampling, leaves, branches and trunk cores were microwaved to halt enzymatic activity. Leaves were microwaved for 2–4 min or until crisp, while branch samples received an additional 1 min of microwaving. Trunk cores were microwaved for 1 min. After enzyme deactivation, samples were oven‐dried at ~60°C in the field before transport for analysis of soluble sugars and starch content.

NSC concentrations were analysed following the protocol of Sevanto et al. (2014) at the Institute of Biology, UNICAMP, Campinas, Brazil. For each sample, 15 mg of dried plant material was ground and mixed with 1.6 mL of distilled water. Starch was enzymatically digested into glucose using amyloglucosidase from *Aspergillus niger* (Sigma‐Aldrich). Low molecular weight sugars (glucose, fructose and sucrose) were quantified using a combination of invertase, glucose hexokinase (GHK) kits and phosphoglucose isomerase (Sigma‐Aldrich). Free glucose concentration was determined photometrically using a 96‐well microplate spectrophotometer (BioTek, Epoch). Starch was calculated as the difference between total NSCs and the concentration of low molecular weight sugars.

### 2.7 Leaf Morphological Traits

Following gas exchange measurements, the leaves used for photosynthetic capacity and *R*_dark_ were collected and stored in airtight ziplock bags with moist cotton wool to maintain humidity. At the end of the day, leaves were scanned using a flatbed scanner (CanoScan LiDE 220; Canon Inc., Tokyo, Japan) and leaf area was determined using ImageJ software (Schneider et al. 2012).

Leaf thickness was measured at three locations on each leaf using digital callipers, avoiding major veins, and an average value was calculated. To ensure full hydration, the branches collected for gas exchange measurements were left overnight in a bucket covered with a black plastic bag. Fully hydrated leaves were then removed, weighed and scanned before being dried at 60°C for 48 h or until a stable weight was reached, before being weighed again. These data were used to calculate branch‐level leaf mass per area (LMA) and leaf dry matter content (LDMC).

The sapwood transversal area of each branch was estimated by measuring branch diameter with digital callipers, which was then used to calculate the leaf area to sapwood area ratio (LA:SA).

### 2.8 Leaf Nutrient Concentrations

Leaves collected for gas exchange and morphological trait measurements were subsequently analysed for carbon ([C]_leaf_), nitrogen ([N]_leaf_), phosphorus ([P]_leaf_), calcium ([Ca]_leaf_), potassium ([K]_leaf_) and magnesium ([Mg]_leaf_) concentrations. Samples were first dehydrated at 80°C for 24 h, then ground to a fine powder (0.2 mm) using an Eberbach E3303 Mini Mill.

Carbon and nitrogen concentrations were determined using the total combustion method with a TURMAC C‐N analyser (Leco, Michigan, USA). Approximately 0.4 g of each sample was weighed into a ceramic crucible and incinerated in a horizontal furnace at 1350°C. All analyses were conducted in triplicate. Carbon content was measured using a non‐dispersive infrared absorption detector, while nitrogen content was quantified using a thermal conductivity detector.

Calcium, potassium and magnesium concentrations were analysed via atomic absorption spectroscopy (AA PinAAcle 900; Perkin Elmer, Massachusetts, USA), while phosphorus concentration was determined colorimetrically using a UV–visible spectrophotometer with a microplate reader (Elx800; Biotek, Vermont, USA), following the malachite green method (D'Angelo et al. 2001). Prior to analysis, samples were digested using the dry ashing method: 1 g of dried, pulverised leaf material was placed in ceramic crucibles and incinerated in a Thermoconcept KLE muffle furnace at 500°C for 4 h. The resulting ash was diluted with 1 N hydrochloric acid and heated in a closed flask at 100°C for 1 h to complete digestion. Samples were analysed at varying dilutions using atomic absorption spectroscopy for [Ca]_leaf_, [K]_leaf_ and [Mg]_leaf_, while [P]_leaf_ concentrations were determined colorimetrically. Calibration curves were generated according to Lambert–Beer's Law to ensure precise quantification. All leaf nutrient analyses were undertaken at the Institution of Nature, Earth and Energy, Pontifical Catholic University of Peru, Lima, Peru.

### 2.9 Data Analysis

#### 2.9.1 Carbon Fluxes

To assess the impact of throughfall exclusion on ecosystem carbon fluxes, we compared 95% confidence intervals between the CON and TFE plots. Confidence intervals were calculated using standard errors, assuming that variation among sampling locations within each treatment follows a normal distribution. Error propagation for all composite variables was conducted using conventional quadrature rules (Hughes and Hase 2010), based on the assumption that uncertainties were independent and normally distributed.

#### 2.9.2 Traits

To evaluate the impact of throughfall exclusion on tree physiological traits, we applied linear mixed‐effects models for each of the 39 measured traits, with treatment as a fixed effect and species nested in genus as a random effect. Species was included as a random effect to allow the treatment effects to be isolated while incorporating taxonomic variation. Additionally, treatment was included as a random slope effect to test whether genera exhibited differential response to throughfall exclusion. Model selection was based on Akaike Information Criterion (AIC) scores, with the best‐fitting model identified as the one with the lowest AIC. To meet assumptions of normality, Ѱ_md_ and *Q*_sat75_ were square‐root transformed, while φ, LCP, R_dark_, g_dark_, LMA, leaf thickness, [Ca]_leaf_ and [K]_leaf_ were natural log‐transformed.

## 3 Results

### 3.1 Carbon Dynamics

A 69.12% reduction of soil moisture on the TFE plot led to a decrease in GPP of 4.24 ± 1.97 Mg C ha^−1^ year^−1^ relative to the CON (Figure 1; Table 1). Autotrophic respiration was also suppressed on the TFE plot by 3.38 ± 1.42 Mg C ha^−1^ year^−1^ relative to the CON. This meant that total NPP was similar on the CON (7.57 ± 0.86 Mg C ha^−1^ year^−1^) and TFE (6.71 ± 1.05 Mg C ha^−1^ year^−1^) despite the large drought‐associated reduction in GPP. The slightly lower total NPP in the TFE compared to the CON was offset by 2.58 ± 1.10 Mg C ha^−1^ year^−1^ lower heterotrophic respiration of soil organic matter (*R*_het_) in the TFE. The end result was that both the CON and TFE acted as a net carbon sink (CON: −1.53 ± 1.18 Mg C ha^−1^ year^−1^; TFE: −1.99 ± 1.14 Mg C ha^−1^ year^−1^). The lower carbon use efficiency (CUE) on the CON (0.304 ± 0.039) was not significantly different from the TFE (0.325 ± 0.055; Table 1). Moreover, trees allocated a similar portion of biomass growth belowground (15.8% ± 20.2%) in the TFE compared to the CON plot (11.9% ± 15.2%).

**TABLE 1:** Ecosystem‐level carbon fluxes of the control (CON) and throughfall exclusion (TFE) plots, including net primary production (NPP) and respiration (R) components.

Flux	CON	TFE
Unit	Mg C ha^−1^ year^−1^	Mg C ha^−1^ year^−1^
Gross primary productivity	24.91 ± 1.46	20.67 ± 1.32
NPP litterfall	3.83 ± 0.41	3.04 ± 0.35
NPP leaf herbivory	0.54 ± 0.41	0.36 ± 0.35
NPP branches	0.49 ± 0.21	0.96 ± 0.63
NPP stems	1.81 ± 0.58	1.29 ± 0.64
NPP coarse roots	0.38 ± 0.12	0.27 ± 0.13
NPP fine roots	0.52 ± 0.09	0.79 ± 0.18
R leaves	6.08 ± 0.32	6.62 ± 0.19
R stems	7.48 ± 0.77	5.91 ± 0.48
R coarse woody debris	0.03 ± 0.01	0.05 ± 0.01
R coarse roots	1.57 ± 0.16	1.24 ± 0.10
R rhizosphere	1.03 ± 0.47	−0.03 ± 0.25
R fine litterfall	0.49 ± 1.19	0.30 ± 0.68
R soil organic matter	6.04 ± 0.80	4.72 ± 0.44
Net ecosystem exchange	−1.53 ± 1.18	−1.99 ± 1.14
Total NPP	7.57 ± 0.86	6.71 ± 1.05
Above‐ground NPP	6.67 ± 0.85	5.65 ± 1.03
Below‐ground NPP	0.90 ± 0.15	1.06 ± 0.22
Autotrophic respiration	17.34 ± 1.17	13.96 ± 0.80
Carbon use efficiency	0.304 ± 0.039	0.325 ± 0.055
Soil respiration	7.57 ± 1.00	4.99 ± 0.46
Total respiration*	23.38 ± 1.42	18.68 ± 0.91

*Note:* Values represent means ± standard errors presented for each plot. Significant differences based on confidence intervals are indicated by asterisks (*p < 0.05). See Table S2 for comparisons of the CON with nearby plots at Esperanza, Wayqecha and Trocha Union III (Malhi et al. 2017).

**FIGURE 2:**
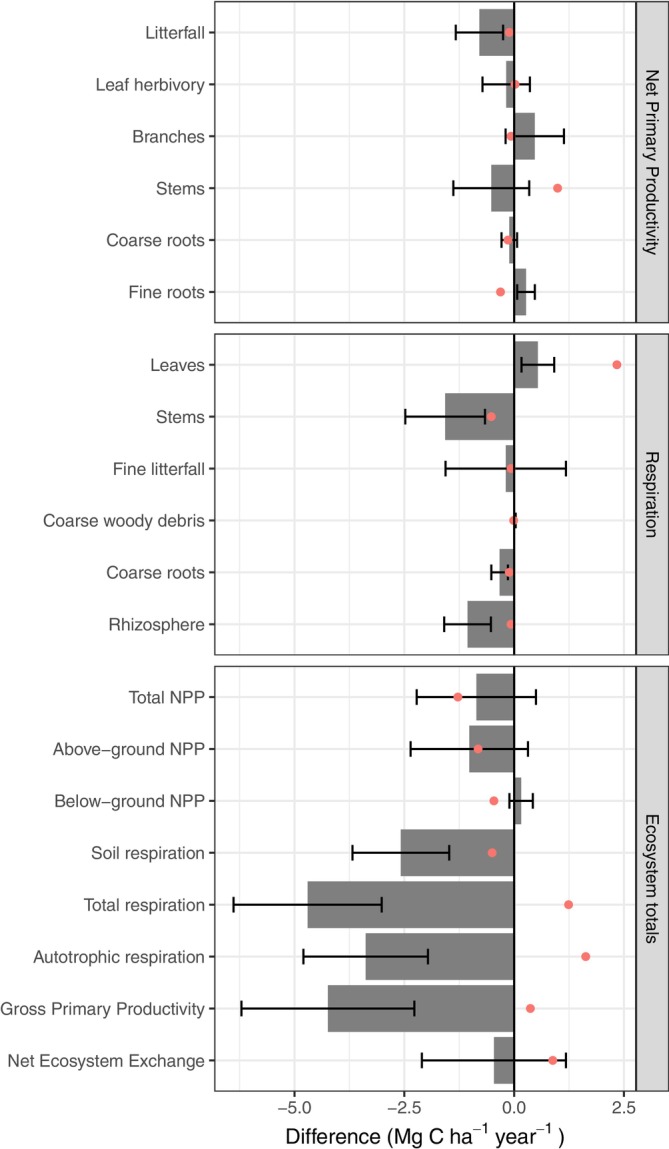
Differences in the net primary production (NPP), respiration and ecosystem totals between a control (CON) and throughfall exclusion experiment (TFE). Positive bars represent components that were larger in the TFE and negative bars represent components that were lower in the TFE compared with CON. Error bars represent standard error of the mean difference; Red dots represent the mean difference in values from a nearby fog reduction experiment (Bartholomew, D. C., Bittencourt, P. R. L., Galiano Cabrera, D., Sacatuma Cruz, R., Asbjornsen, H., Brum, M., Chambi Paucar, J. R., Corrales Alvarez, D., Cosio, E., Espinoza Otazu, B., Mamani, D. M., Meir, P., Muñoz Hermoza, G. A., Oliveira, R. S., Puma Vilca, B. L., Rosalai, A., Salas Yupayccana, C., Salinas, N., Sanchez Tintaya, J., Vadeboncoeur, M. A., Yuca Palomino, J. A., Metcalfe, D. B., unpublished data). For the absolute value of the components, see Table 1.

All net primary production and autotrophic respiration components had overlapping 95% confidence intervals between the CON and TFE plots, indicating that observed shifts were likely non‐significant. Although overall litterfall production did not change between the CON (3.83 ± 0.41 Mg C ha^−1^ year^−1^) and TFE (3.04 ± 0.35 Mg C ha^−1^ year^−1^), the allocation to different canopy components did change substantially under throughfall exclusion. In the TFE experiment, leaf production was 60.0% ± 8.8% lower and fruit production was 90.6% ± 29.8% lower than the CON (Table 2; Figure 2). In contrast, bryophyte production was 47.4% ± 10.9% higher in the TFE compared with the CON (Table 2; Figure 2).

**TABLE 2:** Ecosystem‐level litterfall production of the control (CON) and throughfall exclusion (TFE) plots.

Flux	CON	TFE
Unit	kg C ha^−1^ year^−1^	kg C ha^−1^ year^−1^
Leaves*	3014.62 ± 342.10	1884.56 ± 174.74
Branches	515.01 ± 87.45	586.27 ± 98.15
Barks	17.92 ± 8.63	11.22 ± 3.00
Twigs	532.93 ± 93.56	597.48 ± 99.82
Flowers	31.38 ± 7.31	84.01 ± 23.83
Fruits*	186.82 ± 26.92	98.02 ± 12.60
Bryophytes*	75.81 ± 14.80	144.12 ± 17.31
Bromeliads	0.02 ± 0.02	0.00 ± 0.00
Epiphytes	0.92 ± 0.48	4.07 ± 3.09
Other	1.48 ± 0.46	1.34 ± 0.33

*Note:* Values represent means ± standard errors presented for each plot. Significant differences based on confidence intervals are indicated by asterisks (*p < 0.05).

### 3.2 Tree Physiology

**FIGURE 3:**
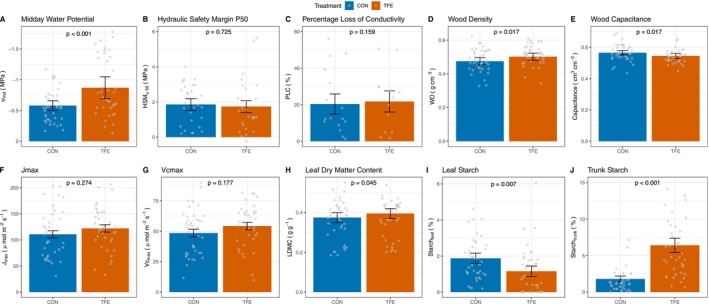
Mean ± standard error of (A) midday leaf water potential (Ѱ_md_), (B) hydraulic safety margin to 50% xylem embolism (HSM_Ѱ50_), (C) percentage loss of xylem conductivity (PLC), (D) wood density (WD), (E) wood capacitance (F) maximum electron conductance rate (*J*_max_), (G) maximum carboxylation rate (*V*_cmax_), (H) leaf dry matter content (LDMC), (I) leaf starch concentration (Starch_leaf_) and (J) trunk starch concentration (Starch_trunk_) in the control (CON) and throughfall exclusion experiment (TFE). *p*‐values represent significance between the two treatments from linear mixed effects models accounting for species nested in genus variation. See Table S1 for more details.

We found that trees had 0.29 ± 0.07 MPa lower Ψ_md_ after 5 years of throughfall exclusion (*p* = 0.06), indicating that changes in soil moisture imposed marginal drought stress on the trees. Whilst this absolute value is small, it represented a 49.9% ± 23.8% reduction on the TFE relative to the CON plot. However, this reduction in Ψ_md_ was insufficient to induce embolism in the trees with no significant change in the percentage loss of conductivity (Δ = 1.43% ± 4.28%; *p* = 0.159), HSM_Ψ50_ (Δ = −0.12 ± 3.32 MPa; *p* = 0.725) or HSM_Ψ88_ (Δ = 0.03 ± 0.48 MPa; *p* = 0.949) between trees in the TFE and the CON (Figure 3; Table S1).

Hydraulic traits largely did not respond to the TFE treatment, with no changes observed in Ψ_50_ (Δ = −0.17 ± 0.30 MPa; *p* = 0.570), Ψ_88_ (Δ = −0.32 ± 0.47 MPa; *p* = 0.489), *k*_smax_ (Δ = −2679.47 ± 3325.73 kg m m^−2^ s^−1^; *p* = 0.429), *k*_sleaf_ (Δ = −8.41 ± 10.08 kg m m^−2^ s^−1^; *p* = 0.841), *g*_datk_ (Δ = −0.005 ± 0.008 mmol m^−2^ s^−1^; *p* = 0.550), leaf hydrophobicity (Δ = −1.63° ± 2.67°; *p* = 0.545) and leaf water retention (Δ = 1.42° ± 1.06°; *p* = 0.182). Changes in wood properties were, however, observed, with higher wood density (Δ = 0.027 ± 0.011 g cm^−3^; *p* = 0.017) and lower wood capacitance (Δ = 0.020 ± 0.008 cm^3^ cm^−3^; *p* = 0.017) on the TFE compared with the CON (Figure S3; Table S1).

Leaf traits did not differ between the CON and TFE, except for LDMC which was higher (Δ = 0.020 ± 0.010 g g^−1^; *p* = 0.045; Figure 3) on the TFE compared to the CON. All measures of leaf photosynthetic capacity and leaf respiration did not significantly differ between the plots (Figure S3; Table S1).

Throughfall exclusion altered non‐structural carbohydrate (NSC) concentrations, with a large increase in total trunk NSC on the TFE compared with the CON (Δ = 4.57% ± 1.12%; *p* = 0.005). This increase in total trunk NSC is explained by a large increase in starch concentrations (Δ = 4.58% ± 1.03%; *p* = 0.003), rather than soluble sugars (Δ = −0.11% ± 0.14%; *p* = 0.452). Non‐structural carbohydrate concentrations in leaves also differed between the CON and TFE, with lower starch concentrations (Δ = −0.71% ± 0.26%; *p* = 0.007). However, this reduction in starch was insufficient to alter total NSC concentrations in leaves between the two plots (Δ = −0.44% ± 0.42%; *p* = 0.295). No change in NSC concentrations was observed in branches (Figure S4).

**FIGURE 4:**
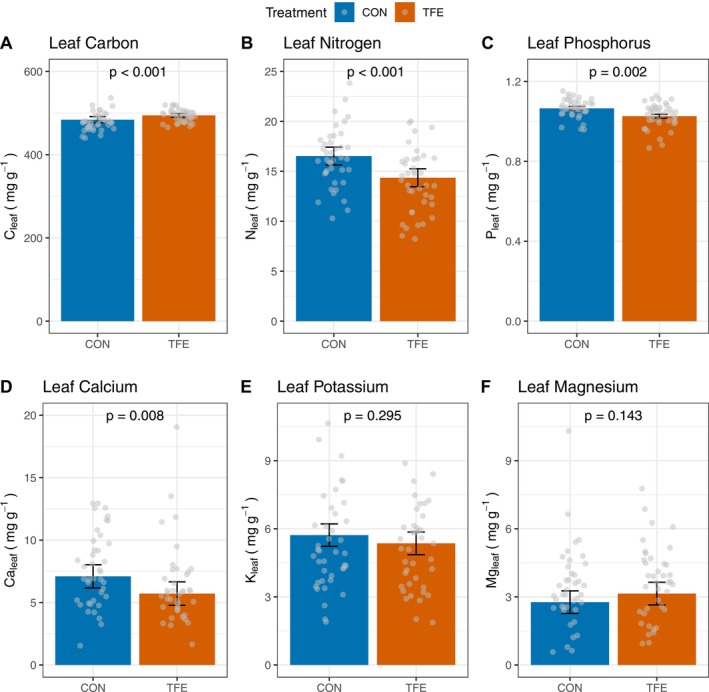
Mean ± standard error of leaf nutrient concentrations in the control (CON) and throughfall exclusion experiment (TFE): (A) carbon, (B) nitrogen, (C) phosphorus, (D) calcium, (E) potassium and (F) magnesium. *p*‐values represent significance between the two treatments from linear mixed effects models accounting for species nested in genus variation. See Table 2 for more details.

Drought conditions also caused a change in leaf nutrient concentrations. In the TFE plot, [N]_leaf_ (Δ = −2171.93 ± 360.18 mg g^−1^; *p* < 0.001), [P]_leaf_ (Δ = −0.039 ± 0.012 mg g^−1^; *p* = 0.002), and [Ca]_leaf_ (Δ = −1.38 ± 0.50 mg g^−1^; *p* = 0.008) were all found to be lower than in the CON plot (Figure 3). In contrast, [K]_leaf_ (Δ = −0.36 ± 0.34 mg g^−1^; *p* = 0.295) and [Mg]_leaf_ (Δ = 0.38 ± 0.26 mg g^−1^; *p* = 0.143) did not change under TFE conditions. Carbon concentrations in leaves did not differ between the TFE and CON (Δ = 10,451 ± 5584 mg g^−1^; *p* = 0.107), although responses varied by species (Figure 4).

**FIGURE 5:**
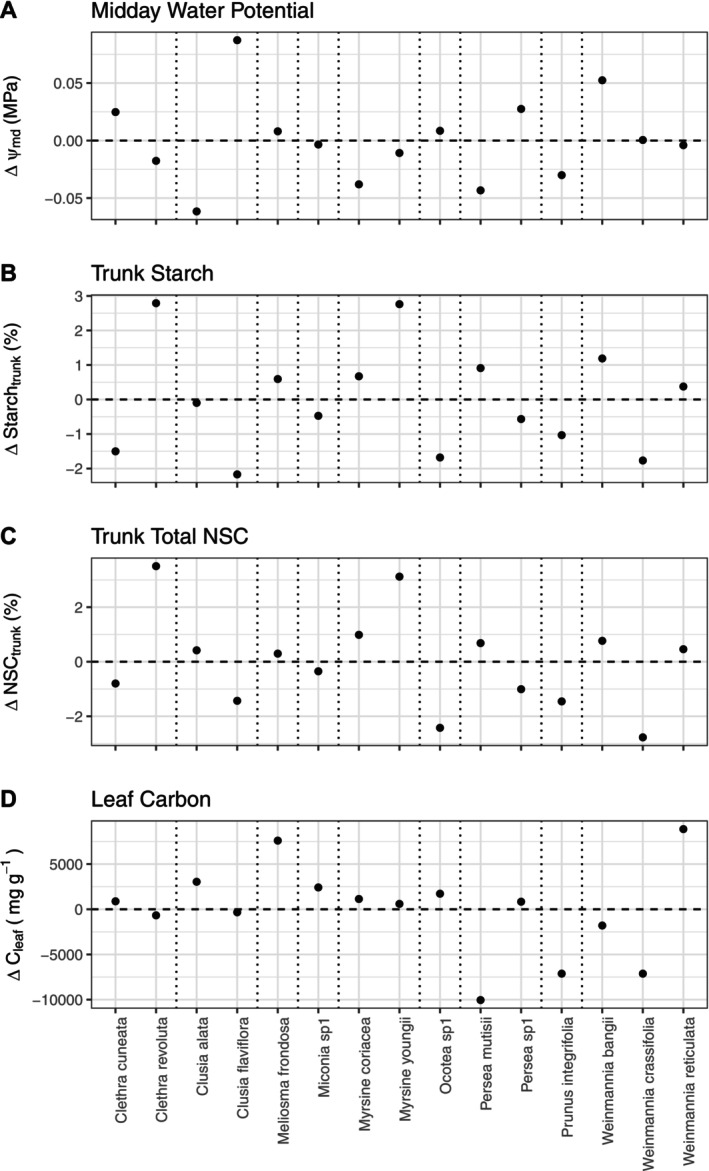
Variation in the mean effect size for 15 species across nine genera to the TFE treatment compared with the CON for (A) midday leaf water potential (Ѱ_md_), (B) trunk starch concentration (Starch_trunk_), (C) total trunk non‐structural carbohydrate concentration (NSC_trunk_) and (D) leaf carbon concentration ([C]_leaf_). Dots represent the difference from the mean treatment effect size, estimated using linear mixed effects models (see Table 2).

The response to the TFE treatment was independent of taxonomic identity for 32 of the 39 traits studied (Table 2). However, four of the 11 traits that had significantly different mean traits values between the CON and TFE showed significant variation in the drought treatment effect among species (Ѱ_md_, Starch_trunk_, NSC_trunk_ and [C]_leaf_). The species that showed the largest difference between the TFE and CON was not consistent across traits. *Clusia alata* had the largest treatment effect for Ѱ_md_, *Clethra revoluta* for Starch_trunk_ and NSC_trunk_, and *Weinmannia reticulata* for [C]_leaf_ (Figure 5). In contrast, the smallest treatment effect size was found for *Clusia flaviflora* for Ѱ_md_ and Starch_trunk_, *Weinmannia crassifolia* for NSC_trunk_ and *Persea mutisii* for [C]_leaf_ (Figure 5).

## 4 Discussion

We examined the effects of throughfall exclusion on ecosystem‐level C dynamics and tree physiological responses in an Andean TMCF. The forest studied displays a remarkable resilience to sustained drought conditions, with both ecosystem C fluxes and individual tree responses remaining largely stable. After 5 years of severely reduced soil moisture, neither NEE nor tree hydraulic status showed significant decline, as would be expected based on responses in lowland Amazonian forests (Bittencourt et al. 2020; Doughty et al. 2015; Rowland, da Costa, et al. 2015). Instead, trees appeared to adopt compensatory strategies, such as accumulating non‐structural carbohydrates in the trunk, increasing branch wood density and producing more conservative leaves. However, these adjustments were accompanied by a reduction in leaf nutrient availability and fruit production under drought, suggesting potential trade‐offs in resource allocation with important long‐term consequences. If the physiological responses of the forest surveyed in this study are representative of TMCFs more widely, the results indicate that TMCFs may be more buffered against long‐term hydrological changes than previously recognised.

### 4.1 Carbon Dynamics

Despite 5 years of severe reductions in throughfall, ecosystem C dynamics remained remarkably stable. Gross primary production (GPP) declined moderately in the TFE plot, reinforcing evidence from the same experiment that found reduced sap flow (Brum et al. 2023) associated with drought. The tighter stomatal control and reduced transpiration in the TFE plot likely drive a reduction in total photosynthetic assimilation and biomass production. Autotrophic respiration showed a similar decline as GPP, meaning NPP in the TFE did not show significant differences from the control. Meanwhile, heterotrophic respiration did not significantly change under drought conditions, meaning NEE was not impacted by drought. This subdued drought response contrasts with more variable drought responses in lowland tropical forests, where drought‐induced reductions in GPP and heterotrophic respiration often occur in the absence of compensatory declines in autotrophic respiration, leading to reduced or destabilised C sink capacity (Brando et al. 2008; Doughty et al. 2015).

While drought conditions generally suppress both photosynthesis (Flexas 2002; Lawson et al. 2010) and heterotrophic respiration (Davidson et al. 2006; Orchard and Cook 1983), autotrophic respiration is often stable or increases, including in lowland tropical forests (Doughty et al. 2015; Metcalfe et al. 2010; Rowland, Da Costa, Oliveira, Oliveira, et al. 2018). Under water deficit conditions, increased demands for maintenance, osmotic adjustment and cellular repair typically increase the use of carbohydrates (Atkin and Macherel 2009; Rowland, Da Costa, Oliveira, Oliveira, et al. 2018), which is further enhanced by the strategy to maintain growth (Rowland, da Costa, et al. 2015). In contrast, we found that trees in TMCF reduced autotrophic respiration proportionally with NPP and do not appear to prioritise growth. This suggests that trees employ a resource‐conservation approach that minimises metabolic demand to avoid long‐term stress. It remains unclear, however, whether this will enable the forest to avoid long‐term biomass loss, or if insufficient time has passed for drought to induce mortality, since biomass losses under drought conditions can be delayed (Rowland, da Costa, et al. 2015).

Resource allocation to reproduction in trees reflects a dynamic balance between current reproductive effort and future survival, especially under resource‐limited conditions (Adler et al. 2014; Chave et al. 2010). Although total litterfall remained unchanged under drought conditions in our experiment, its composition shifted. A decline in fruit production indicates that trees in TMCF prioritise survival over reproduction under drought conditions. Meanwhile, reduced leaf production likely reflects an increase in leaf longevity, consistent with a shift towards a more conservative strategy under drought conditions (Wright et al. 2004). This shift was further supported by an increase in leaf dry matter content under throughfall exclusion. In contrast to a decline in fruit production, flowering showed a marginal, non‐significant increase under drought conditions. This decoupling suggests that trees initiated reproductive development but may have aborted fruit maturation because of carbon or nutrient limitation, prioritising resource retention over reproduction. Early‐stage reproductive failure is consistent with findings from lowland tropical forests, where fruiting initially reduced at a similar drought experiment (Rowland, Da Costa, Oliveira, Almeida, et al. 2018). It remains to be seen, however, whether TMCFs have the capacity to re‐stabilise reproduction over the long term as found in lowland forests (Rowland, Da Costa, Oliveira, Almeida, et al. 2018), especially given the different responses in autotrophic respiration. The decline in reproductive allocation may protect carbon balance initially in the short term but could have major implications for long‐term recruitment and demographic resilience.

In contrast to tree NPP, bryophyte production increased under throughfall exclusion, suggesting that drought‐induced shifts in canopy function and microclimate cascade into the understory. High bryophyte cover and species diversity characterise TMCFs (Horwath et al. 2019), relying on high ambient humidity and regular fog immersion. A nearby fog reduction experiment found bryophyte production to be sensitive to fog (Anders et al. 2025). The increase in bryophyte production here could contribute to accumulation of greater bryophyte biomass, and consequently greater water storage by bryophytes (Slate et al. 2024) in TMCFs under reduced soil moisture conditions. Alternatively, this response could instead reflect greater mortality and/or physical disturbance on the TFE plots. Further work is required to disentangle the C and nutrient dynamics of epiphytes in the TFE experiment, and TCMFs more widely.

### 4.2 Tree Physiological Responses

Hydraulic failure and carbon starvation are widely recognised as the two primary physiological pathways leading to drought‐induced tree mortality (McDowell et al. 2018). Trees on the TFE plot did not show increased risk of hydraulic failure, carbohydrate starvation or reduced metabolic capacity under drought conditions. No significant differences in hydraulic safety margins or xylem conductivity between treatments indicate that the risk of hydraulic failure is not increased after 5 years of soil moisture reduction. Isohydric regulation likely plays a key role in preventing xylem embolism formation, a strategy consistent with observations of reduced gross primary productivity and sap flow (Brum et al. 2023), and increased water use efficiency at the same experiment (Brum et al. 2023). Stomatal closure is likely the dominant response to water deficit given that minimum stomatal conductance, hydraulic conductivity, xylem resistance, leaf area to sapwood area ratios and leaf water retention and hydrophobicity did not respond to the treatment.

Maintenance of hydraulic safety margins and xylem conductivity under the TFE treatment contrasts with observations from lowland tropical forests (Barros et al. 2019; Bittencourt et al. 2020) where drought conditions reduce hydraulic safety margins and increase xylem embolism formation. Larger trees are typically more vulnerable to hydraulic failure (Araújo et al. 2024; Bittencourt et al. 2020; Giles et al. 2022; Rowland, da Costa, et al. 2015) given the additional gravitational potential on the xylem water column. The increased resilience in TMCF trees may therefore be explained by their shorter stature and thus reduced overall tension in the hydraulic pathway. Additionally, the persistence of cloud immersion in TMCFs may buffer trees against drought stress by reducing evaporative demand (Bittencourt et al. 2019) and/or by rehydrating leaves via foliar water uptake (Berry et al. 2019; Eller et al. 2013, 2016). It may also be possible that trees in TMCFs are able to implement embolism repair mechanisms (Klein et al. 2018) or maintain positive xylem pressure (Schenk et al. 2021) to avoid hydraulic failure.

A notable response to the throughfall exclusion was a change in wood properties, with an increase in wood density and decrease in wood capacitance. While high wood density is often correlated with increased embolism resistance across species (Hacke et al. 2001; Markesteijn et al. 2011), our results showed no change in embolism thresholds (Ψ_50_, Ψ_88_). This suggests that the observed change in wood density reflects passive structural changes rather than an active increase in embolism resistance. Higher wood density may simply reflect a reduction in growth rates (Chave et al. 2009). An increase in leaf dry matter content in the TFE further suggests a shift towards a more conservative, slow‐growing physiological strategy (Reich 2014; Wright et al. 2004).

Under drought conditions, trees also showed changes in non‐structural carbohydrate (NSC) storage, with 142% higher NSC concentrations in the trunk in the TFE than the CON plot. The increase in trunk NSC was driven by the accumulation of starch, a metabolically inactive storage form, suggesting trees prioritised carbon retention over growth, respiration and reproduction. Remarkably, this accumulation occurred despite no change in photosynthetic capacity and under reduced GPP. A reduction in autotrophic respiration, leaf and fruit production that all require use of carbohydrates (Würth et al. 2005) will likely have been key to allow NSC stores to increase. The accumulation of NSC likely acts as an important buffer against carbon starvation, ensuring trees avoid mortality from carbon in addition to hydraulic failure (McDowell et al. 2018).

Reduced soil moisture influenced the nutrient cycle, with reduced nitrogen, phosphorus and calcium concentrations observed in leaves after 5 years of throughfall reduction. Reduced sap flow in trees at the site (Brum et al. 2023) likely slows the delivery of nutrients to leaves (Barker and Becker 1995), whilst reductions in microbial and rhizosphere activity under soil drought may have reduced soil nutrient cycling efficiency (Baldrian et al. 2023; Buscardo et al. 2021). Reductions in leaf and fruit litterfall observed under the throughfall exclusion treatment may further reduce the rate of nutrient cycling. These reductions in leaf nutrient concentrations differ from a similar experiment in lowland tropical forests where they remained stable after 15 years of 50% throughfall reduction (Bartholomew et al. 2020; Rowland et al. 2021). Trees in both TMCFs and lowland tropical forests did, however, show some capacity to increase photosynthetic nutrient use efficiency (Bartholomew et al. 2020). The reduction in leaf nutrient concentrations observed here, though, may constrain the potential to increase maximum photosynthetic capacity and the ability for TMCF trees to enhance GPP under drier future conditions.

Despite the diverse species composition in the TMCF plot, most trait responses to drought were remarkably consistent across taxa, showing no significant variation at the species or genus level. However, total trunk NSCs and starch showed considerable interspecific variation in response to the TFE treatment. Given the critical role of NSCs in preventing carbon starvation, this variation may lead to variability in species capacity to survive and/or invest in growth and reproduction over the long term. These differences may affect future demographic trajectories and community composition if long‐term drought conditions persist in TMCFs. Our study adds to the growing evidence that species show highly variable responses to climate change in TMCFs (Barros et al. 2022; Cox et al. 2023; Fadrique et al. 2018; Rehm and Feeley 2016).

### 4.3 Soil Versus Atmospheric Drought

TMCFs receive water inputs from both rainfall‐driven soil moisture and fog water inputs (Bruijnzeel et al. 2011; Goldsmith et al. 2013). While this study focuses on throughfall exclusion and soil moisture drought, a nearby fog reduction experiment (Metcalfe et al. 2025) enables direct comparison to assess how these distinct hydrological inputs affect forest function. From equivalent measurements at the fog reduction experiment (Bartholomew, D. C., Bittencourt, P. R. L., Galiano Cabrera, D., Sacatuma Cruz, R., Asbjornsen, H., Brum, M., Chambi Paucar, J. R., Corrales Alvarez, D., Cosio, E., Espinoza Otazu, B., Mamani, D. M., Meir, P., Muñoz Hermoza, G. A., Oliveira, R. S., Puma Vilca, B. L., Rosalai, A., Salas Yupayccana, C., Salinas, N., Sanchez Tintaya, J., Vadeboncoeur, M. A., Yuca Palomino, J. A., Metcalfe, D. B., unpublished data) and this TFE experiment, noticeable contrasts were detected. Whilst throughfall exclusion suppressed GPP, total NPP and total autotrophic respiration, fog reduction did not alter GPP, suppressed NPP and elevated autotrophic respiration (Bartholomew, D. C., Bittencourt, P. R. L., Galiano Cabrera, D., Sacatuma Cruz, R., Asbjornsen, H., Brum, M., Chambi Paucar, J. R., Corrales Alvarez, D., Cosio, E., Espinoza Otazu, B., Mamani, D. M., Meir, P., Muñoz Hermoza, G. A., Oliveira, R. S., Puma Vilca, B. L., Rosalai, A., Salas Yupayccana, C., Salinas, N., Sanchez Tintaya, J., Vadeboncoeur, M. A., Yuca Palomino, J. A., Metcalfe, D. B., unpublished data). These results indicate different carbon allocation strategies, with soil drought promoting metabolic downregulation and resource conservation, while reductions in fog raise maintenance costs, potentially because of increased evaporation demand, reduced leaf wetness, and altered light conditions (Bartholomew, D. C., Bittencourt, P. R. L., Galiano Cabrera, D., Sacatuma Cruz, R., Asbjornsen, H., Brum, M., Chambi Paucar, J. R., Corrales Alvarez, D., Cosio, E., Espinoza Otazu, B., Mamani, D. M., Meir, P., Muñoz Hermoza, G. A., Oliveira, R. S., Puma Vilca, B. L., Rosalai, A., Salas Yupayccana, C., Salinas, N., Sanchez Tintaya, J., Vadeboncoeur, M. A., Yuca Palomino, J. A., Metcalfe, D. B., unpublished data).

Despite contrasting carbon fluxes, trees did not show hydraulic vulnerability to reductions in either soil moisture or fog reduction, with no changes in hydraulic safety margins or conductivity detected (Figure S5; Bartholomew, D. C., Bittencourt, P. R. L., Galiano Cabrera, D., Sacatuma Cruz, R., Asbjornsen, H., Brum, M., Chambi Paucar, J. R., Corrales Alvarez, D., Cosio, E., Espinoza Otazu, B., Mamani, D. M., Meir, P., Muñoz Hermoza, G. A., Oliveira, R. S., Puma Vilca, B. L., Rosalai, A., Salas Yupayccana, C., Salinas, N., Sanchez Tintaya, J., Vadeboncoeur, M. A., Yuca Palomino, J. A., Metcalfe, D. B., unpublished data). These findings highlight the conservative hydraulic strategies of TMCF species that may be facilitated by their short stature, slow growth rates, lower atmospheric temperatures and multiple water sources (Eller et al. 2020). In contrast to hydraulic responses, carbohydrate strategies did differ among the throughfall and fog reduction treatments, with trees investing in long‐term carbohydrate storage under soil moisture deficit but reducing carbohydrate stores under fog reduction (Bartholomew, D. C., Bittencourt, P. R. L., Galiano Cabrera, D., Sacatuma Cruz, R., Asbjornsen, H., Brum, M., Chambi Paucar, J. R., Corrales Alvarez, D., Cosio, E., Espinoza Otazu, B., Mamani, D. M., Meir, P., Muñoz Hermoza, G. A., Oliveira, R. S., Puma Vilca, B. L., Rosalai, A., Salas Yupayccana, C., Salinas, N., Sanchez Tintaya, J., Vadeboncoeur, M. A., Yuca Palomino, J. A., Metcalfe, D. B., unpublished data). TMCFs therefore appear more vulnerable to carbon starvation under reduced fog than rainfall conditions.

Whilst separating the impacts of reduced fog and rainfall are key to understanding physiological function, climate change is likely to lead to simultaneous alterations in rainfall and fog regimes (Bruijnzeel et al. 2011; Still et al. 1999). However, we are not aware of any study that has tested experimentally their combined effects. Since rainfall likely acts as an important buffer under atmospheric drought, and fog likely acts as an important buffer under soil moisture drought, responses are unlikely to be equivalent if both sources of water input decline at the same time. Indeed, interactive effects from other climate change compound effects can lead to elevated tree mortality (Gazol and Camarero 2022; Kleinman et al. 2019; Yan et al. 2025; Zscheischler et al. 2018). Future work should prioritise factorial drought experiments and trait‐based modelling to identify interactive effects of soil and atmospheric drought in TMCFs, and to predict species and functional groups most at risk. Additionally, drought experiments should be combined with other risk factors, such as changes in temperature and fire, to better predict how environmental change will impact TMCFs.

## Author Contributions

**David C. Bartholomew:** methodology, conceptualization, data curation, investigation, validation, formal analysis, visualization, writing – original draft, writing – review and editing. **Paulo R. L. Bittencourt:** methodology, data curation, formal analysis. **Darcy Galiano Cabrera:** investigation, project administration. **Roxana Sacatuma Cruz:** investigation, project administration. **Jimmy R. Chambi Paucar:** investigation. **Daniela Corrales Alvarez:** investigation. **Eric Cosio:** resources. **Blanca Espinoza Otazu:** investigation. **Darwin Manuel Mamani:** investigation. **Patrick Meir:** conceptualization, resources. **George A. Muñoz Hermoza:** investigation. **Rafael S. Oliveira:** resources. **Beisit L. Puma Vilca:** investigation, project administration. **Aida Rosalai:** investigation. **Carlos Salas Yupayccana:** investigation. **Norma Salinas:** resources. **José Sanchez Tintaya:** investigation. **Jhon A. Yuca Palomino:** investigation. **Daniel B. Metcalfe:** conceptualization, methodology, validation, supervision, funding acquisition, project administration, resources, writing – review and editing.

## Funding

This work was supported by Svenska Forskningsrådet Formas (2023‐00361, 2015‐10002), Vetenskapsrådet (2019‐04779, 201306395) and European Research Council (ECOHERB 682707).

## Conflicts of Interest

The authors declare no conflicts of interest.

## Supporting information


**Figure S1:** gcb70670‐sup‐0001‐FigureS1.pdf.

**Figure S2:** gcb70670‐sup‐0002‐FigureS2.pdf.

**Figure S3:** gcb70670‐sup‐0003‐FigureS3.pdf.

**Figure S4:** gcb70670‐sup‐0004‐FigureS4.pdf.

**Figure S5:** gcb70670‐sup‐0005‐FigureS5.pdf.

**Appendix S1:** gcb70670‐sup‐0006‐AppendixS1.docx.

## Data Availability

The data that support the findings of this study are openly available in FigShare at https://doi.org/10.6084/m9.figshare.29577470.v1 and https://doi.org/10.6084/m9.figshare.29606267.
