# Supplementary figures and images for: Tropical Montane Cloud Forests Have High Resilience to Five Years of Severe Soil Drought
Source: Glob Chang Biol. 2026 Jan 7;32(1):e70670. doi: 10.1111/gcb.70670 (PMC12779095; doi:10.1111/gcb.70670)

Treatment — CON — TFE

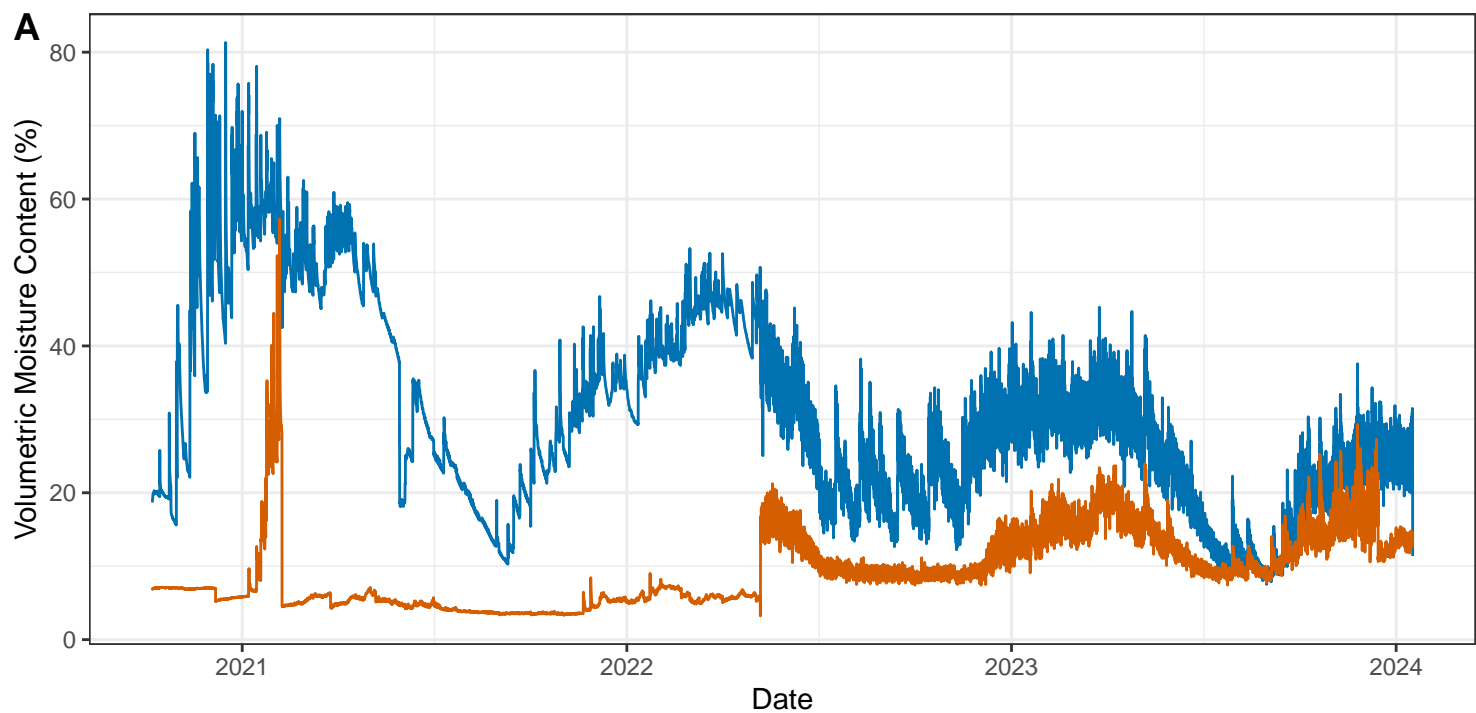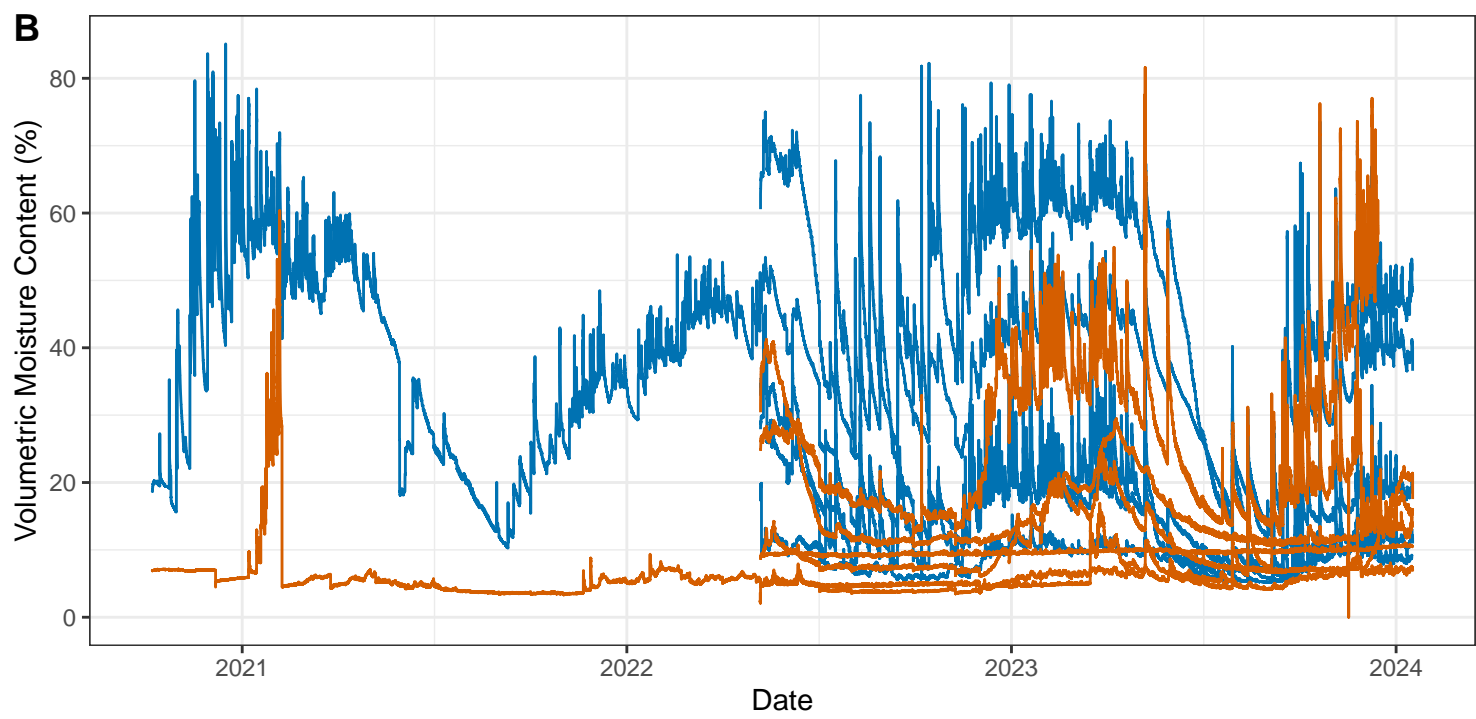

Supplement: Supplementary file 1 — Figure S1: gcb70670‐sup‐0001‐FigureS1.pdf. [file GCB-32-e70670-s006.pdf]

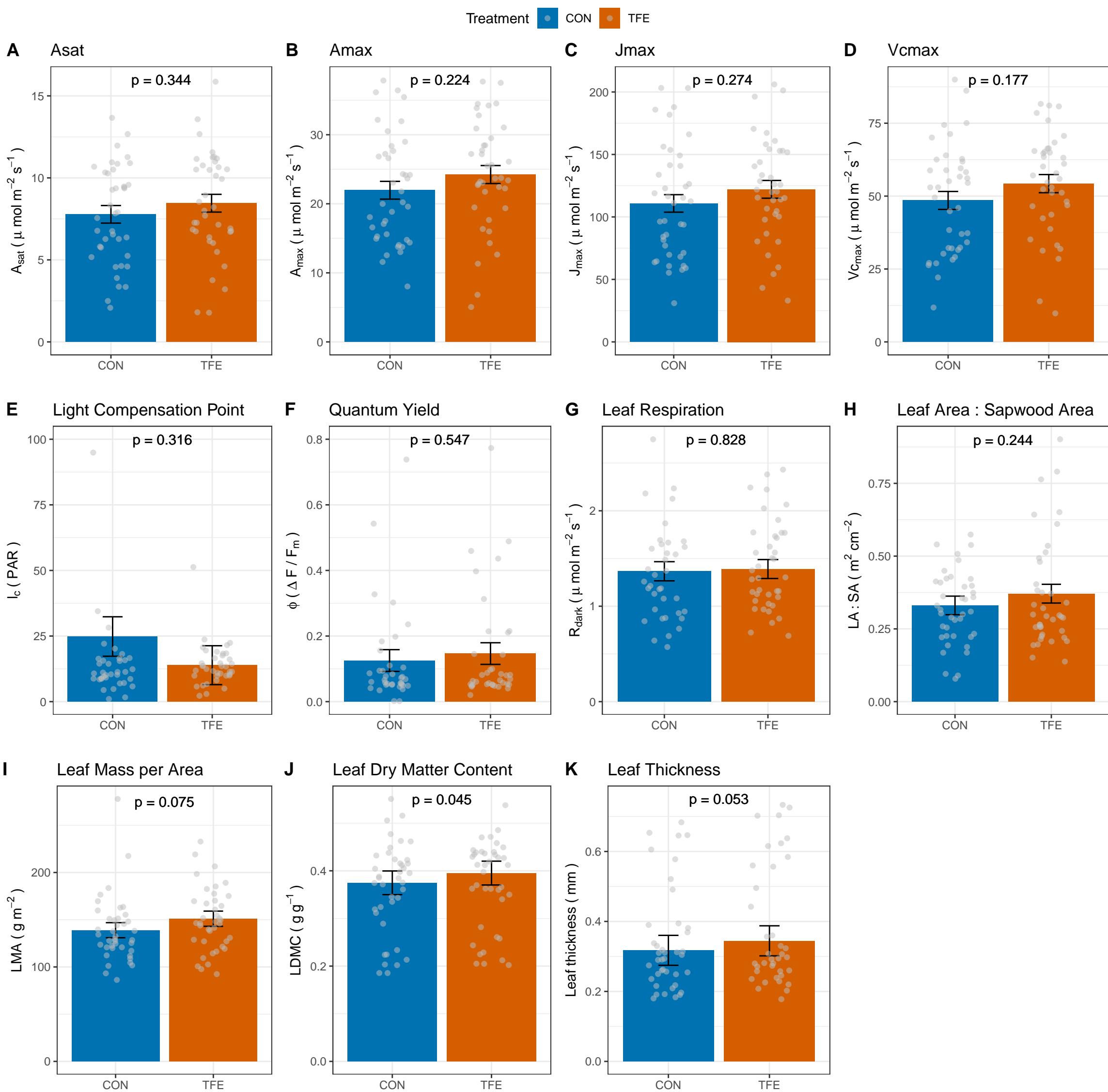

Supplement: Supplementary file 3 — Figure S3: gcb70670‐sup‐0003‐FigureS3.pdf. [file GCB-32-e70670-s004.pdf]

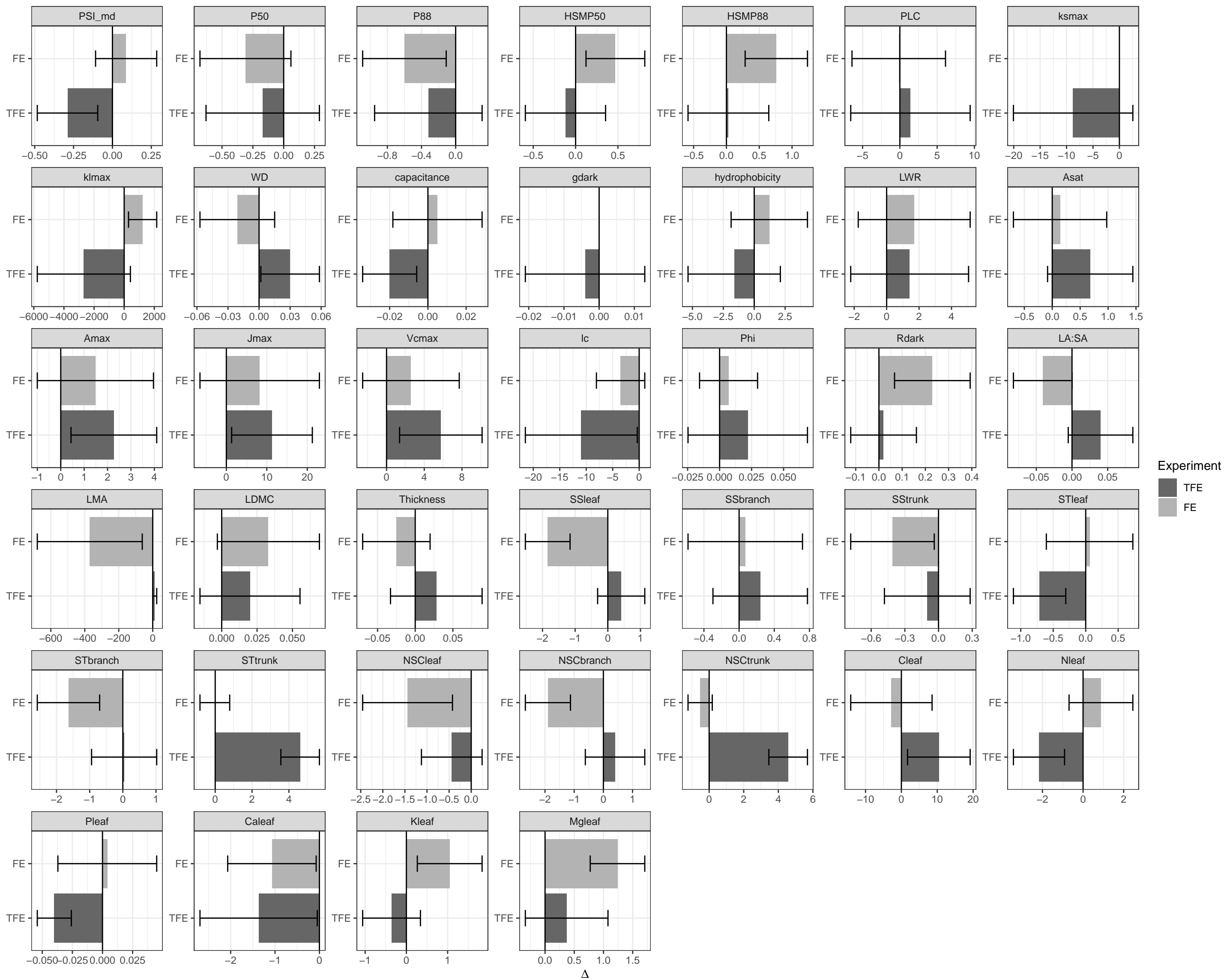

Supplement: Supplementary file 5 — Figure S5: gcb70670‐sup‐0005‐FigureS5.pdf. [file GCB-32-e70670-s005.pdf]
