# Supplementary material for: Tropical Montane Cloud Forests Have High Resilience to Five Years of Severe Soil Drought
Source: Glob Chang Biol. 2026 Jan 7;32(1):e70670. doi: 10.1111/gcb.70670 (PMC12779095; doi:10.1111/gcb.70670)
Supplement: Supplementary file 2 — Figure S2: gcb70670‐sup‐0002‐FigureS2.pdf. [file GCB-32-e70670-s001.pdf]

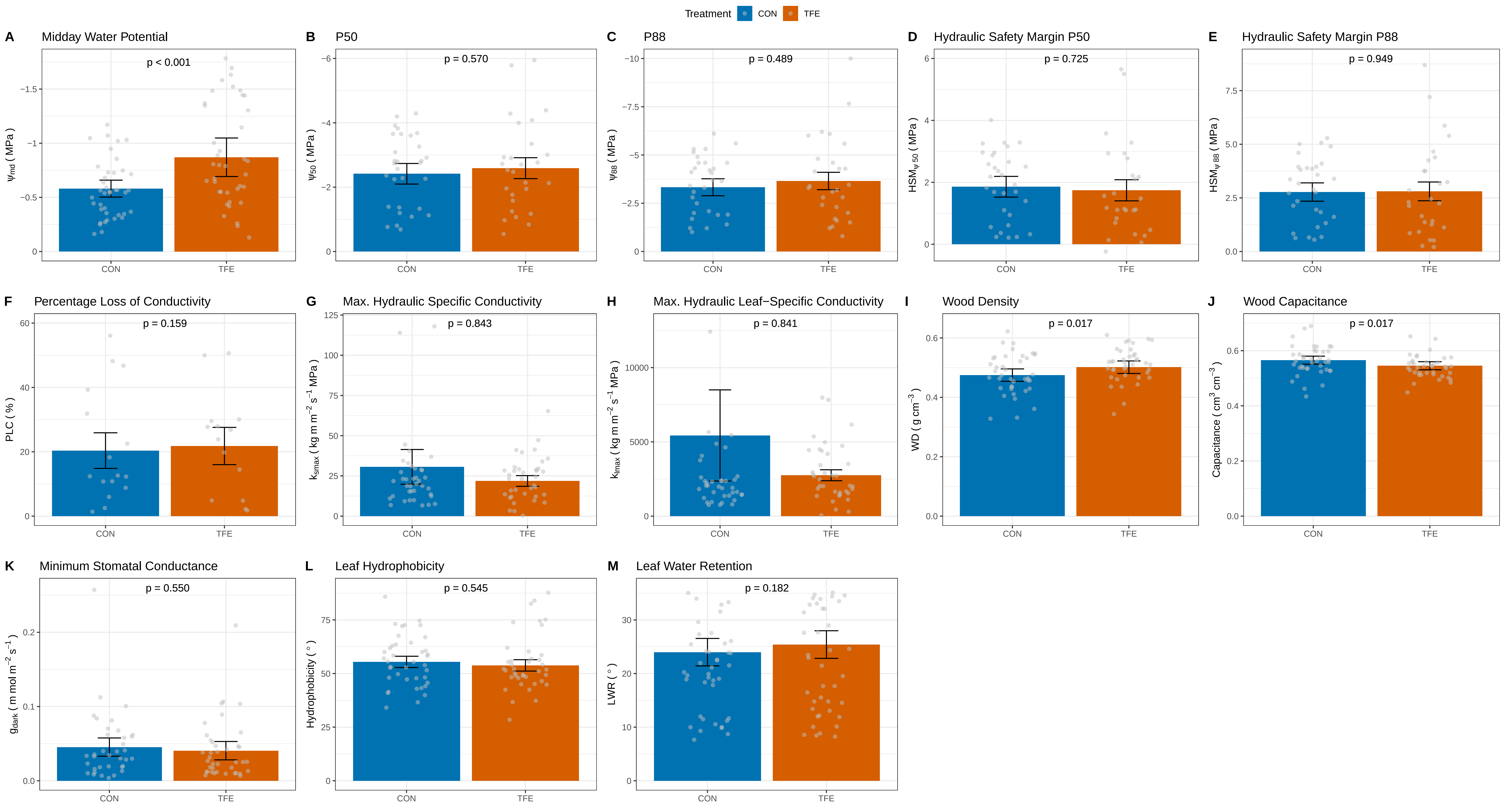
Treatment CON TFE
A Midday Water Potential
p < 0.001
ψmd (MPa)
B P50
p = 0.570
ψ50 (MPa)
C P88
p = 0.489
ψ88 (MPa)
D Hydraulic Safety Margin P50
p = 0.725
HSMψ50 (MPa)
E Hydraulic Safety Margin P88
p = 0.949
HSMψ88 (MPa)
F Percentage Loss of Conductivity
p = 0.159
PLC (%)
G Max. Hydraulic Specific Conductivity
p = 0.843
ksmax (kg m m−2 s−1 MPa)
H Max. Hydraulic Leaf−Specific Conductivity
p = 0.841
klmax (kg m m−2 s−1 MPa)
I Wood Density
p = 0.017
WD (g cm−3)
J Wood Capacitance
p = 0.017
Capacitance (cm3 cm−3)
K Minimum Stomatal Conductance
p = 0.550
gdark (m mol m−2 s−1)
L Leaf Hydrophobicity
p = 0.545
Hydrophobicity (°)
M Leaf Water Retention
p = 0.182
LWR (°)
CON
TFE
