# Supplementary material for: Tropical Montane Cloud Forests Have High Resilience to Five Years of Severe Soil Drought
Source: Glob Chang Biol. 2026 Jan 7;32(1):e70670. doi: 10.1111/gcb.70670 (PMC12779095; doi:10.1111/gcb.70670)
Supplement: Supplementary file 4 — Figure S4: gcb70670‐sup‐0004‐FigureS4.pdf. [file GCB-32-e70670-s002.pdf]

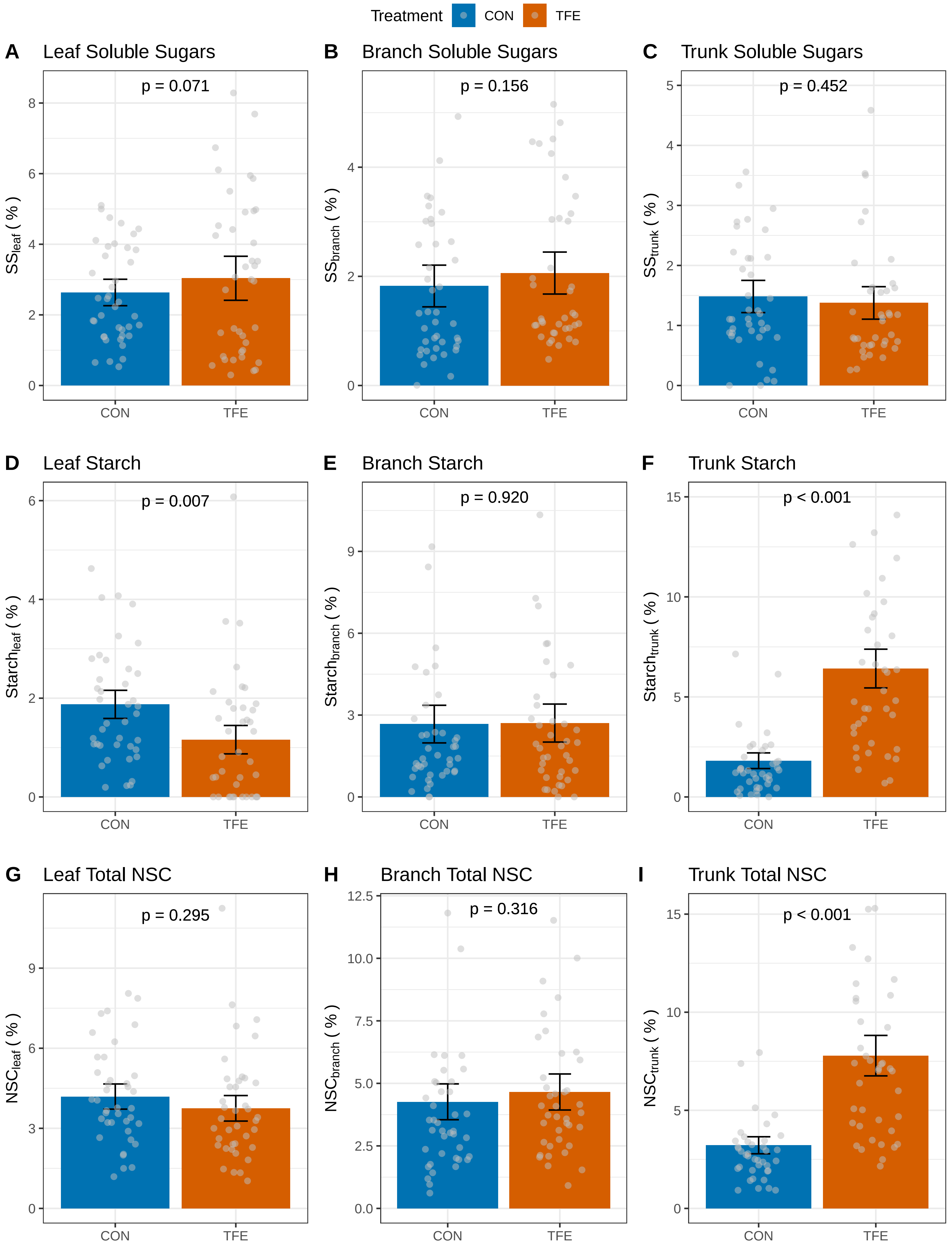

Treatment CON TFE
A Leaf Soluble Sugars
p = 0.071
SSleaf (%)
B Branch Soluble Sugars
p = 0.156
SSbranch (%)
C Trunk Soluble Sugars
p = 0.452
SStrunk (%)
D Leaf Starch
p = 0.007
Starchleaf (%)
E Branch Starch
p = 0.920
Starchbranch (%)
F Trunk Starch
p < 0.001
Starchtrunk (%)
G Leaf Total NSC
p = 0.295
NSCleaf (%)
H Branch Total NSC
p = 0.316
NSCbranch (%)
I Trunk Total NSC
p < 0.001
NSCtrunk (%)
CON TFE
