# Supplementary material for: Tropical Montane Cloud Forests Have High Resilience to Five Years of Severe Soil Drought
Source: Glob Chang Biol. 2026 Jan 7;32(1):e70670. doi: 10.1111/gcb.70670 (PMC12779095; doi:10.1111/gcb.70670)
Supplement: Supplementary file 6 — Appendix S1: gcb70670‐sup‐0006‐AppendixS1.docx. [file GCB-32-e70670-s003.docx]

# Supplementary Information

**SI Figure 1:** Water availability on the control (CON) and throughfall exclusion experiment (TFE): (a) mean volumetric water content across sensors; (b) mean volumetric content for five sensors in the CON and six sensors in the TFE. Initially only one sensor was installed per plot before additional sensors were installed on 07 May 2022. The additional sensors increased the spatial coverage within the CON and TFE plots, creating a greater variability in soil moisture measurements. The TFE had a 69.1% reduced volumetric moisture content when averaging the mean across sensors across the whole study period.

**SI Figure 2**: Mean ± standard error of hydraulic traits in the control (CON) and throughfall exclusion experiment (TFE): (a) midday leaf water potential (Ѱ_md_), (b) water potential at 50% xylem embolism (Ѱ_50_), (c) water potential at 88% xylem embolism (Ѱ_88_), (d) hydraulic safety margin to 50% xylem embolism (HSM_Ѱ50_), (e) hydraulic safety margin to 88% xylem embolism (HSM_Ѱ50_) (f) percentage loss of xylem conductivity (PLC), (g) maximum hydraulic specific conductivity (k_smax_), (h) maximum hydraulic leaf-specific conductivity (k_sleaf_), (i) wood density (WD), (j) wood capacitance, (k) minimum stomatal conductance in the dark (g_dark_), (l) leaf hydrophobicity and (m) leaf water retention (LWR). P-values represent significance between the two treatments from linear mixed effects models accounting for species nested in genus variation. See Table 2 for more details.

**SI Figure 3**: Mean ± standard error of leaf gas exchange and physiological traits in the control (CON) and throughfall exclusion experiment (TFE): (a) light saturated photosynthetic rate (A_sat_), (b) maximum photosynthetic rate (A_max_), (c) maximum electron conductance rate (J_max_), (d) maximum carboxylation rate (Vc_max_), (e) light compensation point (l_c_), (f) photosynthetic quantum yield (ɸ), (g) dark-adapted leaf respiration (R_dark_), (h) leaf area to sapwood area ratio (LA : SA), (i) leaf mass per area (LMA), (j) leaf dry matter content (LDMC), and (k) leaf thickness. P-values represent significance between the two treatments from linear mixed effects models accounting for species nested in genus variation. See Table 2 for more details.

**SI Figure 4**: Mean ± standard error of non-structural carbohydrate (NSC) concentrations in the control (CON) and throughfall exclusion experiment (TFE): (a) leaf soluble sugars (SS_leaf_), (b) branch soluble sugars (SS_branch_), (c) trunk soluble sugars (SS_trunk_), (d) leaf starch (Starch_leaf_), (e) branch starch (Starch_branch_), (f) trunk starch (Starch_trunk_), (g) total leaf NSC (NSC_leaf_), (h) total branch NSC (NSC_branch_), and (i) total trunk NSC (NSC_trunk_). P-values represent significance between the two treatments from linear mixed effects models accounting for species nested in genus variation. See Table 2 for more details.

**SI Figure 5:** Differences in trait values between a control and treatment for a throughfall exclusion experiment (TFE) and a fog reduction experiment (FE). Positive bars represent components that were larger in the treatment and negative bars represent components that were lower in the treatment compared with the control. Error bars represent standard error of the mean difference. PSI_md: Midday Water Potential; P50: Water Potential at 50% xylem embolism; P88: Water Potential at 88% xylem embolism; HSMP50: Hydraulic Safety Margin to 50% xylem embolism; HSMP88: Hydraulic Safety Margin to 88% xylem embolism; PLC: Percentage loss of hydraulic conductivity; ksmax: Maximum hydraulic specific conductivity; klmax: Maximum hydraulic leaf-specific conductivity; WD: Wood density; capacitance: Wood capacitance; gdark: Minimum stomatal conductance; hydrophobicity: Leaf hydrophobicity; LWR: Leaf Water Retention; Asat: Light saturated photosynthetic rate; Amax: Maximum photosynthetic rate; Jmax: Maximum electron conductance rate; Vcmax: Maximum carboxylation rate; lc: Light Compensation Point; Phi:Photosynthetic quantum yield; Rdark: Dark-adapted leaf respiration; LA:SA: Leaf Area to Sapwood Area ratio; LMA: Leaf Mass per Area; LDMC: Leaf Dry Matter Content; Thickness: Leaf thickness; SSleaf: Leaf Soluble Sugars; SSbranch: Branch Soluble Sugars; SStrunk: Trunk Soluble Sugars; STleaf: Leaf Starch; STbranch: Branch Starch; STtrunk: Trunk Starch; NSCleaf: Total leaf non-structural carbohydrates; NSCbranch: Total branch non-structural carbohydrates; NSCtrunk: Total trunk non-structural carbohydrates; Cleaf: Leaf Carbon; Nleaf: Leaf Nitrogen; Pleaf: Leaf Phosphorus; Caleaf: Leaf Calcium; Kleaf: Leaf Potassium; Mgleaf: Leaf Magnesium. For the absolute value of the components and units, see SI Table 1.

**SI Table 1:** Mean **±** standard error of 39 leaf and wood traits for the control (CON) and throughfall exclusion (TFE) plots. Values represent parameter estimates from mixed effects models with species nested in genus as a random intercept variable. Species and Genus represent the standard deviation for these variables, respectively. For traits where the AIC score for the random slope effect was lower, the standard deviation of the random slope effect is presented for species (Species | TFE) and genus (Genus | TFE). P-values represent significant values for the model with the random intercept, whilst R^2^_total_ and R^2^_fixed_ represent the variance explained for the model including random effects and the plot treatment only, respectively.

| **Trait** | | **Unit** | **n** | **CON** | **TFE** | **Species** | **Species \| TFE** | **Genus** | **Genus \| TFE** | **p** | **R^2^_total_** | **R^2^_fixed_** |
| --- | --- | --- | --- | --- | --- | --- | --- | --- | --- | --- | --- | --- |
| Midday Water Potential | Ѱ_md_ | MPa | 79 | -0.581 ± 0.078 | -0.870 ± 0.178 | 0.01 | 0.10 | 0.20 | 0.29 | 0.064 | 0.747 | 0.095 |
| Water Potential at 50% xylem embolism | Ѱ_50_ | MPa | 57 | -2.42 ± 0.32 | -2.59 ± 0.33 | 0.34 | - | 0.61 | - | 0.570 | 0.298 | 0.005 |
| Water Potential at 88% xylem embolism | Ѱ_88_ | MPa | 57 | -3.33 ± 0.44 | -3.65 ± 0.45 | 0.40 | - | 0.77 | - | 0.489 | 0.218 | 0.008 |
| Hydraulic Safety Margin to 50% xylem embolism | HSM_Ѱ50_ | MPa | 57 | 1.86 ± 0.33 | 1.74 ± 0.34 | 0.33 | - | 0.64 | - | 0.725 | 0.279 | 0.002 |
| Hydraulic Safety Margin to 88% xylem embolism | HSM_Ѱ88_ | MPa | 57 | 2.77 ± 0.43 | 2.80 ± 0.44 | 0.40 | - | 0.68 | - | 0.949 | 0.174 | 0.000 |
| Percentage loss of hydraulic conductivity | PLC | % | 30 | 20.37 ± 5.54 | 21.79 ± 5.79 | 6.11 | - | 12.43 | - | 0.742 | 0.644 | 0.002 |
| Maximum hydraulic specific conductivity | k_smax_ | kg m m^-2^ s^-1^ MPa | 75 | 30.62 ± 10.80 | 21.86 ± 3.33 | 0.00 | 8.68 | 31.82 | 27.71 | 0.429 | 0.860 | 0.027 |
| Maximum hydraulic leaf-specific conductivity | k_lmax_ | kg m m^-2^ s^-1^ MPa | 75 | 5432.65 ± 3069.18 | 2753.17 ± 368.05 | 7631.83 | 8000.72 | 6330.29 | 6176.14 | 0.445 | 0.954 | 0.033 |
| **Wood density** | **WD** | **g cm^-3^** | **79** | **0.47 ± 0.02** | **0.50 ± 0.02** | **0.01** | **-** | **0.06** | **-** | **0.017** | **0.625** | **0.032** |
| **Wood capacitance** |  | **cm^3^ cm^-3^** | **79** | **0.57 ± 0.01** | **0.55 ± 0.01** | **0.02** | **-** | **0.04** | **-** | **0.017** | **0.593** | **0.035** |
| Minimum stomatal conductance | g_dark_ | m mol m^-2^ s^-1^ | 75 | 0.045 ± 0.012 | 0.041 ± 0.012 | 0.00 | - | 0.03 | - | 0.550 | 0.487 | 0.003 |
| Leaf hydrophobicity |  | ° | 79 | 55.42 ± 2.63 | 53.80 ± 2.65 | 4.71 | - | 3.62 | - | 0.545 | 0.220 | 0.004 |
| Leaf Water Retention | LWR | ° | 79 | 23.98 ± 2.56 | 25.40 ± 2.58 | 2.04 | - | 7.06 | - | 0.182 | 0.737 | 0.007 |
| Light saturated photosynthetic rate | A_sat_ | μ mol m^-2^ s^-1^ | 77 | 7.78 ± 0.54 | 8.46 ± 0.54 | 0.61 | - | 0.36 | - | 0.344 | 0.064 | 0.012 |
| Maximum photosynthetic rate | A_max_ | μ mol m^-2^ s^-1^ | 77 | 21.96 ± 1.29 | 24.23 ± 1.30 | 0.80 | - | 0.00 | - | 0.224 | 0.031 | 0.020 |
| Maximum electron conductance rate | J_max_ | μ mol m^-2^ s^-1^ | 77 | 110.82 ± 7.01 | 122.10 ± 7.09 | 2.38 | - | 0.00 | - | 0.274 | 0.020 | 0.017 |
| Maximum carboxylation rate | Vc_max_ | μ mol m^-2^ s^-1^ | 77 | 48.52 ± 3.08 | 54.28 ± 3.11 | 3.71 | - | 0.00 | - | 0.177 | 0.065 | 0.025 |
| Light Compensation Point | l_c_ | μ mol photons m^-2^ s^-1^ | 75 | 24.82 ± 7.52 | 13.89 ± 7.42 | 0.00 | - | 1.97 | - | 0.316 | 0.016 | 0.014 |
| Photosynthetic quantum yield | ɸ | ΔF / F_m_ | 73 | 0.125 ± 0.033 | 0.147 ± 0.033 | 0.00 | - | 0.06 | - | 0.547 | 0.161 | 0.005 |
| Dark-adapted leaf respiration | R_dark_ | μ mol m^-2^ s^-1^ | 75 | 1.37 ± 0.10 | 1.39 ± 0.10 | 0.13 | - | 0.16 | - | 0.828 | 0.177 | 0.001 |
| Leaf Area to Sapwood Area ratio | LA : SA | m^2^ cm^-2^ | 79 | 0.331 ± 0.032 | 0.371 ± 0.032 | 0.05 | - | 0.05 | - | 0.244 | 0.182 | 0.016 |
| Leaf Mass per Area | LMA | g m^-2^ | 79 | 138.93 ± 7.98 | 151.07 ± 8.05 | 9.82 | - | 16.64 | - | 0.075 | 0.339 | 0.031 |
| **Leaf Dry Matter Content** | **LDMC** | **g g^-1^** | **79** | **0.375 ± 0.025** | **0.395 ± 0.025** | **0.00** | **-** | **0.07** | **-** | **0.045** | **0.740** | **0.015** |
| Leaf thickness |  | mm | 78 | 0.317 ± 0.043 | 0.345 ± 0.043 | 0.02 | - | 0.12 | - | 0.053 | 0.827 | 0.010 |
| Leaf Soluble Sugars | SS_leaf_ | g g^-1^ | 79 | 2.63 ± 0.37 | 3.04 ± 0.62 | 0.36 | 0.13 | 0.94 | 0.76 | 0.361 | 0.658 | 0.015 |
| Branch Soluble Sugars | SS_branch_ | g g^-1^ | 79 | 1.82 ± 0.38 | 2.06 ± 0.38 | 0.00 | - | 1.08 | - | 0.156 | 0.705 | 0.009 |
| Tunk Soluble Sugars | SS_trunk_ | g g^-1^ | 77 | 1.48 ± 0.27 | 1.38 ± 0.27 | 0.00 | - | 0.73 | - | 0.452 | 0.601 | 0.003 |
| **Leaf Starch** | **Starch_leaf_** | **g g^-1^** | **79** | **1.87 ± 0.28** | **1.16 ± 0.29** | **0.38** | **-** | **0.55** | **-** | **0.007** | **0.074** | **0.328** |
| Branch Starch | Starch_branch_ | g g^-1^ | 79 | 2.67 ± 0.69 | 2.71 ± 0.70 | 0.00 | - | 1.88 | - | 0.920 | 0.582 | 0.000 |
| **Trunk Starch** | **Starch_trunk_** | **g g^-1^** | **77** | **1.81 ± 0.39** | **6.42 ± 0.97** | **0.70** | **1.97** | **0.00** | **1.43** | **0.004** | **0.693** | **0.366** |
| Total leaf non-structural carbohydrates | NSC_leaf_ | g g^-1^ | 79 | 4.19 ± 0.47 | 3.75 ± 0.48 | 0.00 | - | 1.06 | - | 0.295 | 0.270 | 0.011 |
| Total branch non-structural carbohydrates | NSC_branch_ | g g^-1^ | 79 | 4.26 ± 0.72 | 4.66 ± 0.72 | 0.90 | - | 1.79 | - | 0.316 | 0.607 | 0.006 |
| **Total trunk non-structural carbohydrates** | **NSC_trunk_** | **g g^-1^** | **77** | **3.22 ± 0.43** | **7.79 ± 1.03** | **0.57** | **2.25** | **0.63** | **1.81** | **0.005** | **0.730** | **0.358** |
| Leaf Carbon | [C]_leaf_ | mg g^-1^ | 79 | 484.07 ± 7.56 | 494.52 ± 4.36 | 7766.45 | 8900.83 | 20562.83 | 9210.32 | 0.107 | 0.713 | 0.059 |
| **Leaf Nitrogen** | **[N]_leaf_** | **mg g^-1^** | **79** | **16.53 ± 0.89** | **14.36 ± 0.89** | **2314.71** | **-** | **1715.76** | **-** | **<0.001** | **0.811** | **0.102** |
| **Leaf Phosphorus** | **[P]_leaf_** | **mg g^-1^** | **79** | **1.07 ± 0.01** | **1.03 ± 0.01** | **0.02** | **-** | **0.00** | **-** | **0.002** | **0.219** | **0.116** |
| **Leaf Calcium** | **[Ca]_leaf_** | **mg g^-1^** | **79** | **7.09 ± 0.93** | **5.72 ± 0.94** | **0.39** | **-** | **2.51** | **-** | **0.008** | **0.608** | **0.042** |
| Leaf Potassium | [K]_leaf_ | mg g^-1^ | 79 | 5.72 ± 0.49 | 5.36 ± 0.50 | 0.00 | - | 1.25 | - | 0.295 | 0.428 | 0.009 |
| Leaf Magnesium | [Mg]_leaf_ | mg g^-1^ | 79 | 2.77 ± 0.50 | 3.14 ± 0.50 | 0.70 | - | 1.23 | - | 0.143 | 0.644 | 0.011 |

**SI Table 2:** Ecosystem level carbon fluxes of the control (CON) and three nearby Global Ecosystem Monitoring (GEM) plots. Values represent means ± standard errors presented for each plot. Values from the CON are taken from this study and for Esperanza, Wayqecha and Trocha Union III from Malhi et al., (2017). Respiration fluxes were not measured at Trocha Union III. All fluxes are measured in Mg C ha^-1^ yr^-1^.

| **Flux** | **CON** | **Esperanza** | **Wayqecha** | **Trocha Union III** |
| --- | --- | --- | --- | --- |
| Elevation (m asl) |  | 2863 | 3045 | 3044 |
| Gross Primary Productivity | 24.91 ± 1.46 | 21.76 ± 2.57 | 25.93 ± 3.10 | 17.23 ± 3.30 |
| NPP Litterfall | 3.83 ± 0.41 | 2.94 ± 0.28 | 3.99 ± 0.28 | 2.66 ± 0.01 |
| NPP Leaf herbivory | 0.54 ± 0.41 | 0.25 ± 0.04 | 0.32 ± 0.02 | 0.23 ± 0.01 |
| NPP Branches | 0.49 ± 0.21 | 0.75 ± 0.07 | 0.54 ± 0.05 | 0.41 ± 0.06 |
| NPP Stems | 1.81 ± 0.58 | 2.17 ± 0.22 | 1.18 ± 0.12 | 1.02 ± 0.10 |
| NPP Coarse roots | 0.38 ± 0.12 | 0.46 ± 0.07 | 0.25 ± 0.04 | 0.21 ± 0.03 |
| NPP Fine roots | 0.52 ± 0.09 | 1.41 ± 0.21 | 1.90 ± 0.35 | 1.31 ± 0.23 |
| R Leaves | 6.08 ± 0.32 | 6.10 ± 1.92 | 5.18 ± 1.63 |  |
| R Stems | 7.48 ± 0.77 | 4.87 ± 1.54 | 7.69 ± 2.42 |  |
| R Coarse roots | 1.57 ± 0.16 | 1.02 ± 0.52 | 1.61 ± 0.81 |  |
| R Rhizosphere | 1.03 ± 0.47 | 2.71 ± 0.36 | 3.42 ± 0.50 |  |
| Total NPP | 7.57 ± 0.86 | 7.73 ± 0.42 | 7.86 ± 0.47 | 5.61 ± 0.26 |
| Carbon Use Efficiency | 0.304 ± 0.039 | 0.36 ± 0.05 | 0.30 ± 0.04 | 0.33 ± 0.07 |

## Supplementary Methods

### Carbon Fluxes

NPP_ACW_ was estimated by monitoring stem diameter growth in all trees with a diameter ≥ 10 cm at 1.3 m height within both plots. Each tree was fitted with a dendrometer at this height, and diameter increments were recorded at regular intervals using calipers. Trees were identified to species or genus level where possible. Canopy height estimates followed an allometric equation developed for Moist West Amazonian forests (Feldpausch et al., 2011). Biomass accumulation was calculated using species-specific wood density values and an allometric equation incorporating tree diameter, height and wood density values (Malhi et al., 2006). Biomass was then converted to carbon using a conversion factor of 0.473 (Martin & Thomas, 2011).

Branch turnover (NPP_branch turnover_) was assessed by surveying fallen branches >2 cm in diameter, excluding those originating from standing dead trees. Two permanent transects were established in each plot (1 x 30 m), where branch dimensions were measured to estimate volume. Wood density values were assigned based on decomposition classes (Harmon et al., 1995) and surveys were conducted every three months.

Litterfall (NPP_litterfall_) was measured using nine litter traps (50 x 50 cm) installed at 1 m above the ground, or above the plastic panels in the TFE. Litterfall was collected biweekly, sorted into categories (leaves, woody material, fruits, flowers, seeds) dried at 60 °C to constant mass, and weighed. Carbon content was estimated using a conversion factor of 0.492, based on regional estimates for Amazonian litterfall (Girardin et al., 2014). To account for biomass loss due to herbivory, which would otherwise lead to underestimation of leaf NPP, herbivory losses (NPP_herbivory_) were quantified following (Metcalfe et al., 2014). Leaves collected from litter traps were scanned, and the missing leaf area was estimated using ImageJ software (ImageJ, Maryland, USA).

Belowground NPP was calculated as the sum of coarse root (NPP_coarse roots_) and fine root (NPP_fine roots_) productivity. Coarse root productivity could not be directly measured and was instead estimated as 21% ± 3% of NPP_ACW_, based on scaling relationships from previous studies (Cairns et al., 1997; Jackson et al., 1996). Fine root productivity was assessed using nine ingrowth cores per plot, consisting of root-free soil enclosed in mesh cylinders (12 cm diameter, 30 cm depth). Cores were extracted every three months, root-free soil was replaced, and the cores were reinstalled. Extracted roots were manually separated from soil over four 10-minute intervals to correct for underestimation due to difficult-to-extract roots (Metcalfe et al., 2007). Following separation, roots were rinsed, dried at 60 °C to constant mass, and weighed. Carbon content was estimated using the same conversion factor as NPP_ACW_ (0.473).

Canopy respiration (R_leaves_) was estimated by measuring leaf dark respiration (R_dark_) from canopy leaves collected from 79 trees. A community-weighted mean was calculated for each plot. To account for variations in canopy structure, leaf area index (LAI) was manually every 3-4 months at nine locations per plot using an LAIPen (Photon System Instruments, Drásov, Czech Republic). Plot-level R_leaves_ was estimated by multiplying the community weighted mean R_dark_ by the mean LAI of each plot.

Aboveground live wood respiration (R_stems_) was measured using an infra-red gas analyzer (IRGA; EGM-5 IRGA and SRC-1 chamber, PP Systems, Hitchin, UK). A respiration chamber was attached to permanent collars (12 cm diameter) installed at 1.3 m height on selected trees. Measurements were taken for two minutes, with the respiration rate averaged over the second minute following a one-minute equilibration period. 21 trees in the CON plot and 20 trees in the TFE plot were monitored. To estimate plot-level R_stems_, individual tree respiration values were scaled based on the surface area index, following the approach of (Chambers et al., 2004). Measurements were conducted every three months.

Total soil CO_2_ efflux (R_soil_) was measured monthly at nine locations per plot using an IRGA connected to permanent soil collars (12 cm diameter) inserted to approximately 2 cm into the soil. The same protocol applied to R_stems_ measurements was used for R_soil_. Soil temperature (T260 probe, Testo Ltd, Hampshire, U.K.) and moisture content (Hydrosense probe, Campbell Scientific Ltd, Loughborough, UK) were recorded at 30 cm depth at each measurement point to account for environmental influences on soil CO_2_ fluxes. To partition soil respiration into autotrophic (R_rhizosphere_) and heterotrophic (R_soil_het_) components, three additional collars were installed in each plot corner under different conditions: intact soil with litter, soil with litter and roots removed, and just soil. These treatments allowed for differentiation between root respiration and contributions from microbial decomposition of litter, mycorrhizal activity and soil organic matter decomposition.

### Hydraulic Traits

#### Midday Water Potential

Midday leaf water potential (Ψ_md_) was measured immediately after collection using a Scholander’s pressure chamber (Model 1000, PMS Instrument Company, Albany, Oregon, USA). Branches were sampled between 11:30 and 13:30, and water potential was measured on three leaves per sample. To minimise seasonal biases, sampling days were alternated between the CON and TFE plots.

#### Wood Density, Wood Capacitance, Leaf Hydrophobicity and Leaf Water Retention

Wood density (WD) and wood capacitance were assessed on branch segments measuring 5-20 mm in diameter and 50-100 mm in length. Bark was removed, and the segments were fully rehydrated by submersion in water for 24 hours. Saturated wood volume was determined using the water displacement method (Pérez-Harguindeguy et al., 2013) and saturated mass was measured using a precision balance. Samples were then oven-dried at 60 °C for 48 hours or until reaching a stable weight. Wood capacitance was calculated as the ratio of water mass to saturated mass: (saturated mass – dry mass)/saturated mass.

Leaf hydrophobicity was measured by placing a 10 µl water droplet on two horizontally positioned leaves and photographing it with a macro-lens digital camera (Olympus EM5 Mark II with M.Zuiko Digital ED 60mm f/2.8 Macro lens, Olympus, UK). Contact angles were measured in ImageJ (Schneider et al., 2012), and mean values were calculated. Leaf water retention was determined by placing a 50 µl droplet on the same leaves and gradually tilting them until the droplet began to move. The tilt angle was recorded using a digital protractor.

#### Hydraulic conductance and native embolism

Native and maximum hydraulic conductance were measured by determining branch xylem conductivity before and after emboli removal. Branches (1.5 - 3 m long) were collected at midday, transported 30 minutes to the lab in dark plastic bags with moist tissue and sequentially cut underwater to release tension and prevent artifacts (Venturas et al., 2015). Mean vessel length, determined from 50 branches was 66.4 ± 32.3 cm, suggesting minimal embolism artifacts in branches of this length.

Final segments (1 - 5 cm long, 2.57-10.99 mm in sapwood diameter) were cut underwater, and both ends were freshly cut with a sharp razor blade. These segments were connected to a hydraulic apparatus to measure water flow and pressure across the sample, following (Bittencourt et al., 2022). Water flow was recorded every second using two flowmeters (Sensirion SLF3S-0600F) while pressure was monitored using a pressure transducer (Honeywell 26PCBFA6D) connected to a custom-built data logger. Conductance was calculated as the ratio of flow rate to water pressure.

Native conductivity (K_snat_) was measured by applying ~5 kPa pressure to degassed distilled water for 60 seconds. Maximum conductivity (K_smax_) was determined after flushing emboli from the branch with ~100kPa pressure, followed by another 60-second measurement at 5kPa. The transversal area of each sample was estimated using digital calipers, measuring at diameters at two perpendicular points. Native embolism (PLCnat) was calculated as the percentage loss of conductivity: PLCnat = (1 - K_snat_ / K_smax_) x 100. Maximum leaf specific conductance (K_sleaf_) was derived by dividing K_smax_ by the distal leaf area, estimated from dry leaf mass multiplied by leaf mass per area (LMA).

#### Xylem embolism resistance and hydraulic safety margins

To assess xylem embolism resistance, the relationship between percentage loss of conductance and xylem water potential was quantified using the pneumatic method (Pereira et al., 2016, 2020). Branches (1-2 m long) were collected from the upper canopy between 09:00 and 11:00 and transported 30 minutes to the lab in dark plastic bags containing moist tissue. After two underwater cuts, branches were covered with dark plastic bags and left to rehydrate in buckets overnight.

The following morning, branches were connected to a pneumatron, which measured air discharge at 30-minute intervals to monitor embolism formation (Pereira et al., 2020). Embolism was induced using the bench dehydration method (Sperry et al., 1988), and measured xylem water potential was recorded using a pressure chamber (Model 1505D, PMS Instrument Company, Albany, Oregon, USA) after allowing branches to equilibrate with attached leaves for one hour. Water potential values were interpolated between air discharge measurements (Pereira et al., 2020), and the water potential at 50 % (Ψ_50_) and 88 % (Ψ_88_) air discharge was determined by fitting a sigmoidal curve to the data (Pammenter & Van der Willigen, 1998).

Hydraulic safety margins (HSM_Ψ50_ and HSM_Ψ88_) were calculated to assess drought vulnerability. These were determined as the difference between Ψ_md_ and Ψ_50_ and Ψ_88_, respectively, providing insight into the buffer against hydraulic failure under changing moisture conditions.

### Gas exchange measurements

Gas exchange measurements were conducted on branches collected from the sun-exposed portion of the canopy or, when necessary, from the upper crown. Branches (~1-2 m in length) were cut between 08:00 and 09:30 hours and immediately re-cut twice underwater in buckets to restore hydraulic flow (Domingues et al., 2010). They were then transported in water for ~30 minutes to the Wayqecha Cloud Forest Biological Station, Peru, where they were re-cut twice underwater to maintain water supply to the leaves. After transport, branches were left to stabilize in sunlight for at least 30 minutes before measurement.

Two non-senescing, fully expanded leaves per branch were selected - one for photosynthetic capacity and another for dark-adapted leaf respiration (*R*_dark_). Measurements were performed using two cross-calibrated portable photosynthesis systems (LI-6400XT, LI-COR, Nebraska, USA).

#### Photosynthetic Capacity

Photosynthetic parameters were measured by conducting CO_2_-response (*A-C_i_*) and light-response (*A-Q*) curves. A single leaf was placed in the leaf chamber of the portable photosynthesis system under controlled conditions: a CO_2_ concentration of 410 µmol mol^-1^, photosynthetic active radiation (PAR) of 2000 µmol m^-2^ s^-1^, temperature of 20 °C, and relative humidity of 60-80 %. The PAR level of 2000 µmol m^-2^ s^-1^ was selected based on prior light-response curve saturation points for each genus.

For *A-C_i_* curves, net photosynthetic assimilation (*A*) and leaf internal CO_2_ concentration (*C_i_*) were recorded while adjusting reference CO_2_ concentrations across nine setpoints (410, 300, 200, 150, 50, 410, 800, 1200, 2000 µmol mol^-1^). For *A-Q* curves, *A* was measured while adjusting light quantum flux (*Q*) to ten setpoints (2000, 1800, 1500, 1200, 900, 600, 300, 150, 50, 0 µmol m^-2^ s^-1^ *PAR*). Before recording measurements, steady-state conditions were established (1-2 minutes), and three to five consecutive measurements were taken at 5-second intervals for each setpoint.

The maximum rate of photosynthesis (*A*_max_) and assimilation under saturating light and ambient CO_2_ (*A*_sat_) were determined using the first and last measurements of the *A*-*C_i_* curve. The maximum rate of carboxylation (*V*_cmax_) and the maximum electron transfer rate (*J*_max_) were standardized to 25 °C by fitting (Farquhar et al., 1980) photosynthesis model following (Sharkey et al., 2007), using the *optim* function in R. Light compensation point (*l_c_*) and quantum yield (*Φ*) were estimated by fitting a non-rectangular hyperbola model to the *A*-*Q* curve using the *onls* package (Spiess, 2022) in R, following the equations of (de Lobo et al., 2013).

#### Leaf Dark Respiration (R_dark_)

Leaf respiration in the dark (*R_dark_*) was measured on an adjacent leaf to the one used for photosynthetic capacity. Leaves were dark-adapted by wrapping them in aluminum foil for at least 30 minutes before measurement. *R_dark_* was recorded using the portable photosynthesis system under the following chamber conditions: CO_2_ concentration of 410 µmol mol^-1^, PAR of 0 µmol m^-2^ s^-1^, temperature of 20 °C, and relative humidity of 60-80 %. Measurements were taken after steady-state conditions were reached with five readings at 5-second intervals.

Respiration values were standardized to 25 °C using a Q_10_ value of 2.2, following (Rowland et al., 2015). Stomatal conductance during *R*_dark_ measurements was also recorded to estimate minimum stomatal conductance (*g*_dark_).

Bittencourt, P. R. de L., Bartholomew, D. C., Banin, L. F., Bin Suis, M. A. F., Nilus, R., Burslem, D. F. R. P., & Rowland, L. (2022). Divergence of hydraulic traits among tropical forest trees across topographic and vertical environment gradients in Borneo. *New Phytologist*, *235*, 2183–2198. https://doi.org/10.1111/nph.18280

Cairns, M. A., Brown, S., Helmer, E. H., & Baumgardner, G. A. (1997). Root biomass allocation in the world’s upland forests. *Oecologia*, *111*, 1–11. https://doi.org/10.1007/s004420050201

Chambers, J. Q., Tribuzy, E. S., Toledo, L. C., Crispim, B. F., Higuchi, N., Santos, J. dos, Araújo, A. C., Kruijt, B., Nobre, A. D., & Trumbore, S. E. (2004). Respiration from a Tropical Forest Ecosystem: Partitioning of Sources and Low Carbon Use Efficiency. *Ecological Applications*, *14*(sp4), 72–88. https://doi.org/10.1890/01-6012

de Lobo, F. A., de Barros, M. P., Dalmagro, H. J., Dalmolin, Â. C., Pereira, W. E., de Souza, É. C., Vourlitis, G. L., & Rodríguez Ortíz, C. E. (2013). Fitting net photosynthetic light-response curves with Microsoft Excel—A critical look at the models. *Photosynthetica*, *51*(3), 445–456. https://doi.org/10.1007/s11099-013-0045-y

Domingues, T. F., Meir, P., Feldpausch, T. R., Saiz, G., Veenendaal, E. M., Schrodt, F., Bird, M., Djagbletey, G., Hien, F., Campaore, H., Diallo, A., Grace, J., & Lloyd, J. (2010). Co‐limitation of photosynthetic capacity by nitrogen and phosphorus in West Africa woodlands. *Plant, Cell & Environment*, *33*, 959–980. https://doi.org/10.1111/j.1365-3040.2010.02119.x

Farquhar, G. D., von Caemmerer, S., & Berry, J. A. (1980). A biochemical model of photosynthetic CO2 assimilation in leaves of C3 species. *Planta*, *149*, 78–90. https://doi.org/10.1007/BF00386231

Feldpausch, T. R., Banin, L., Phillips, O. L., Baker, T. R., Lewis, S. L., Quesada, C. A., Affum-Baffoe, K., Arets, E. J. M. M., Berry, N. J., Bird, M., Brondizio, E. S., de Camargo, P., Chave, J., Djagbletey, G., Domingues, T. F., Drescher, M., Fearnside, P. M., França, M. B., Fyllas, N. M., … Lloyd, J. (2011). Height-diameter allometry of tropical forest trees. *Biogeosciences*, *8*(5), 1081–1106. https://doi.org/10.5194/bg-8-1081-2011

Girardin, C. A. J., Farfan-Rios, W., Garcia, K., Feeley, K. J., Jørgensen, P. M., Murakami, A. A., Cayola Pérez, L., Seidel, R., Paniagua, N., Fuentes Claros, A. F., Maldonado, C., Silman, M., Salinas, N., Reynel, C., Neill, D. A., Serrano, M., Caballero, C. J., La Torre Cuadros, M. de los A., Macía, M. J., … Malhi, Y. (2014). Spatial patterns of above-ground structure, biomass and composition in a network of six Andean elevation transects. *Plant Ecology & Diversity*, *7*(1–2), 161–171. https://doi.org/10.1080/17550874.2013.820806

Harmon, M. E., Whigham, D. F., Sexton, J., & Olmsted, I. (1995). Decomposition and Mass of Woody Detritus in the Dry Tropical Forests of the Northeastern Yucatan Peninsula, Mexico. *Biotropica*, *27*(3), 305–316. https://doi.org/10.2307/2388916

Jackson, R. B., Canadell, J., Ehleringer, J. R., Mooney, H. A., Sala, O. E., & Schulze, E. D. (1996). A global analysis of root distributions for terrestrial biomes. *Oecologia*, *108*(3), 389–411. <https://doi.org/10.1007/BF00333714>

Malhi, Y., Girardin, C. A. J., Goldsmith, G. R., Doughty, C. E., Salinas, N., Metcalfe, D. B., Huaraca Huasco, W., Silva‐Espejo, J. E., Del Aguilla‐Pasquell, J., Farfán Amézquita, F., Aragão, L. E. O. C., Guerrieri, R., Ishida, F. Y., Bahar, N. H. A., Farfan‐Rios, W., Phillips, O. L., Meir, P., & Silman, M. (2017). The variation of productivity and its allocation along a tropical elevation gradient: A whole carbon budget perspective. *New Phytologist*, *214*(3), 1019–1032. https://doi.org/10.1111/nph.14189

Malhi, Y., Wood, D., Baker, T. R., Wright, J., Phillips, O. L., Cochrane, T., Meir, P., Chave, J., Almeida, S., Arroyo, L., Higuchi, N., Killeen, T. J., Laurance, S. G., Laurance, W. F., Lewis, S. L., Monteagudo, A., Neill, D. A., Vargas, P. N., Pitman, N. C. A., … Vinceti, B. (2006). The regional variation of aboveground live biomass in old-growth Amazonian forests. *Global Change Biology*, *12*(7), 1107–1138. https://doi.org/10.1111/j.1365-2486.2006.01120.x

Martin, A. R., & Thomas, S. C. (2011). A Reassessment of Carbon Content in Tropical Trees. *PLOS ONE*, *6*(8), e23533. https://doi.org/10.1371/journal.pone.0023533

Metcalfe, D. B., Asner, G. P., Martin, R. E., Silva Espejo, J. E., Huaraca Huasco, W., Farfán Amézquita, F. F., Carranza-Jimenez, L., Galiano Cabrera, D. F., Durand Baca, L., Sinca, F., Huaraca Quispe, L. P., Alzamora Taype, I., Eguiluz Mora, L., Rozas Dávila, A., Mamani Solórzano, M., Puma Vilca, B. L., Laupa Román, J. M., Guerra Bustios, P. C., Salinas Revilla, N., … Malhi, Y. (2014). Herbivory makes major contributions to ecosystem carbon and nutrient cycling in tropical forests. *Ecology Letters*, *17*, 324–332. https://doi.org/10.1111/ele.12233

Metcalfe, D. B., Meir, P., Aragão, L. E. O. C., Malhi, Y., da Costa, A. C. L., Braga, A., Gonçalves, P. H. L., de Athaydes, J., de Almeida, S. S., & Williams, M. (2007). Factors controlling spatio‐temporal variation in carbon dioxide efflux from surface litter, roots, and soil organic matter at four rain forest sites in the eastern Amazon. *Journal of Geophysical Research*, *112*, G04001. https://doi.org/10.1029/2007JG000443

Pammenter, N. W., & Van der Willigen, C. (1998). A mathematical and statistical analysis of the curves illustrating vulnerability of xylem to cavitation. *Tree Physiology*, *18*(8–9), 589–593. https://doi.org/10.1093/treephys/18.8-9.589

Pereira, L., Bittencourt, P. R. L., Oliveira, R. S., Junior, M. B. M., Barros, F. V., Ribeiro, R. V., & Mazzafera, P. (2016). Plant pneumatics: Stem air flow is related to embolism – new perspectives on methods in plant hydraulics. *New Phytologist*, *211*, 357–370. https://doi.org/10.1111/nph.13905

Pereira, L., Bittencourt, P. R. L., Pacheco, V. S., Miranda, M. T., Zhang, Y., Oliveira, R. S., Groenendijk, P., Machado, E. C., Tyree, M. T., Jansen, S., Rowland, L., & Ribeiro, R. V. (2020). The Pneumatron: An automated pneumatic apparatus for estimating xylem vulnerability to embolism at high temporal resolution. *Plant, Cell & Environment*, *43*, 131–142. https://doi.org/Plant Cell Environ

Pérez-Harguindeguy, N., Díaz, S., Garnier, E., Lavorel, S., Poorter, H., Jaureguiberry, P., Bret-Harte, M. S., Cornwell, W. K., Craine, J. M., Gurvich, D. E., Urcelay, C., Veneklaas, E. J., Reich, P. B., Poorter, L., Wright, I. J., Ray, P., Enrico, L., Pausas, J. G., Vos, A. C. de, … Cornelissen, J. H. C. (2013). New handbook for standardised measurement of plant functional traits worldwide. *Australian Journal of Botany*, *61*(3), 167–234. https://doi.org/10.1071/BT12225

Rowland, L., Lobo‐do‐Vale, RMalhi, Y., Girardin, C. A. J., Goldsmith, G. R., Doughty, C. E., Salinas, N., Metcalfe, D. B., Huaraca Huasco, W., Silva‐Espejo, J. E., Del Aguilla‐Pasquell, J., Farfán Amézquita, F., Aragão, L. E. O. C., Guerrieri, R., Ishida, F. Y., Bahar, N. H. A., Farfan‐Rios, W., Phillips, O. L., Meir, P., & Silman, M. (2017). The variation of productivity and its allocation along a tropical elevation gradient: A whole carbon budget perspective. *New Phytologist*, *214*(3), 1019–1032. https://doi.org/10.1111/nph.14189

. L., Christoffersen, B. O., Melém, E. A., Kruijt, B., Vasconcelos, S. S., Domingues, T., Binks, O. J., Oliveira, A. A. R., Metcalfe, D., Da Costa, A. C. L., Mencuccini, M., & Meir, P. (2015). After more than a decade of soil moisture deficit, tropical rainforest trees maintain photosynthetic capacity, despite increased leaf respiration. *Global Change Biology*, *21*(12), 4662–4672. https://doi.org/10.1111/gcb.13035

Schneider, C. A., Rasband, W. S., & Eliceiri, K. W. (2012). NIH Image to ImageJ: 25 years of image analysis. *Nature Methods*, *9*(7), 671–675. https://doi.org/10.1038/nmeth.2089

Sharkey, T. D., Bernacchi, C. J., Farquhar, G. D., & Singsaas, E. L. (2007). Fitting photosynthetic carbon dioxide response curves for C3 leaves. *Plant, Cell & Environment*, *30*, 1035–1040. https://doi.org/10.1111/j.1365-3040.2007.01710.x

Sperry, J. S., Donnelly, J. R., & Tyree, M. T. (1988). A method for measuring hydraulic conductivity and embolism in xylem. *Plant, Cell & Environment*, *11*, 35–40. https://doi.org/10.1111/j.1365-3040.1988.tb01774.x

Spiess, A.-N. (2022). *onls: Orthogonal Nonlinear Least-Squares Regression* (Version 0.1-2) [Computer software]. https://cran.r-project.org/web/packages/onls/index.html

Venturas, M. D., Mackinnon, E. D., Jacobsen, A. L., & Pratt, R. B. (2015). Excising stem samples underwater at native tension does not induce xylem cavitation. *Plant, Cell & Environment*, *38*(6), 1060–1068. https://doi.org/10.1111/pce.12461
